# The effects of sunlight exposure on mortality: a systematic review of epidemiological studies

**DOI:** 10.3310/nihropenres.13980.1

**Published:** 2025-06-18

**Authors:** Thomas Parkhouse, Francesca Spiga, Lesley E Rhodes, Sarah Dawson, Katie E Webster, Deborah M Caldwell, Julian P T Higgins

**Affiliations:** 1 NIHR Bristol Evidence Synthesis Group, Population Health Sciences, Bristol Medical School, University of Bristol, Bristol, England, BS8 2PS, UK; 2 Division of Musculoskeletal and Dermatological Sciences, School of Biological Sciences,, The University of Manchester, Manchester, England, M13 9PL, UK; 3 Dermatology Research Centre, Salford Royal Hospital, Manchester Academic Health Science Centre, Northern Care Alliance NHS Foundation Trust, Salford, England, UK; 4 NIHR Applied Research Collaboration West (ARC West), University Hospitals Bristol and Weston NHS Foundation Trust, Bristol, England, UK

**Keywords:** Systematic review; Sunlight; Ultraviolet radiation; Mortality; Cancer; Cardiovascular disease

## Abstract

**Introduction:**

Current sun safety advice focuses on minimizing exposure to sunlight, due to the relationship between ultraviolet radiation and skin cancer. However, sunlight also has beneficial effects, and there are calls for guidance to reflect these alongside the harmful effects. To examine the net effect of harmful and beneficial aspects, we aimed to determine the association between sunlight exposure and all-cause mortality. Additionally, we examined cause-specific mortality and whether the associations varied according to skin type/colour or ethnicity.

**Methods:**

We conducted a systematic review, searching MEDLINE, Embase, Web of Science and the Cochrane Central Register of Controlled Trials (Nov 2023) for reports of epidemiological studies in the general population investigating the effect of any measure of long-term sun exposure on all-cause, cardiovascular-related, or cancer-related mortality. We conducted a narrative synthesis of the findings and assessed risk of bias using the ROBINS-E tool. PROSPERO: CRD42023474157.

**Results:**

The search identified 73 eligible articles. Methods of measuring sunlight exposure comprised radiation (e.g., ultraviolet radiation levels), proxy measures of radiation (e.g., latitude) and behaviour associated with sunlight exposure (e.g., frequency of sunbathing). The evidence was inconclusive. While most studies of skin cancer mortality found a higher risk associated with more exposure to sunlight, many studies of other cancers reported lower risks associated with more exposure to sunlight. Evidence for all-cause mortality was mixed, as were findings for cardiovascular mortality. Results were subject to high risk of bias, largely due to the likelihood of uncontrolled confounding and the use of indirect measures of sunlight exposure. There were insufficient data regarding any differential effects of sunlight on mortality for those of different skin types/colours or ethnicity.

**Conclusion:**

Findings from observational epidemiological studies of the association between sunlight exposure and mortality are too variable to provide a strong rationale for changes to sun protection guidance.

## Plain Language Summary

**Title:**

The effect of sunlight on death rate: a systematic review of the evidence

**What is the problem?:**

Sunlight causes damage to our skin. Being sunburnt can increase the chance of getting skin cancer. Therefore, sun safety advice in the UK is mainly focused on avoiding the harmful impacts of sunlight. Organizations providing advice on sun exposure say to stay in the shade between 11 am and 3 pm and wear covering clothing.

There are also positive effects of sunlight exposure such as vitamin D production in your body. Vitamin D is important for your bones. Vitamin D or other effects of sunlight may also help reduce your risk of developing some cancers and cardiovascular disease.

People of different skin types react to sunlight in different ways. People with darker skin need more exposure to sunlight than people with lighter skin to produce the same amount of vitamin D, and they also have less risk of developing sun-related skin cancer.

**What did we do?:**

We gathered all the available studies that have measured both sunlight exposure and mortality (death) rates. We were interested in the overall number of deaths (by any cause), as well as deaths specifically caused by cardiovascular disease or cancer.

**What did we find?:**

Our findings are mixed. Exposure to sunlight has been reported both to increase and to decrease your risk of dying. Alongside its harmful effect on skin cancer, sunlight may help prevent other types of cancer. However, there were problems in how the studies were done, so we can’t be certain about the findings. From the information available, there is not strong enough evidence to alter sun exposure advice.

## Introduction

Current sun safety advice is primarily focused on minimizing exposure to sunlight. For example, in the United Kingdom (UK) this includes Cancer Research UK recommendations to keep to the shade between 11 am and 3 pm and to cover up with clothes ^1^, advice that is echoed by the National Health Service (NHS) ^2^ and the National Institute for Health and Care Excellence (NICE) ^3^, and in the USA by the American Academy of Dermatology (AAD) guidance on sunlight exposure ^4^. The reason behind this advice is the well-established relationship between exposure to ultraviolet (UV) radiation (UVR) and skin cancer ^5^. It has been estimated that 86% of melanoma cases are attributable to UVR ^6^, and the risk of developing basal cell carcinoma (BCC), the most common form of non-melanoma skin cancer (NMSC), is 2.12 times greater with every five sunburns experienced as an adult ^7^. Cutaneous squamous cell carcinoma (cSCC), which is capable of metastasizing, is the other common type of NMSC, and shows an increasing incidence in the UK and various European countries ^8, 9^.

There are calls to alter sun exposure advice ^10^. The harmful effects of sunlight exposure should be considered alongside any beneficial effects. For example, sunlight exposure is usually the body’s main source of vitamin D ^11^, which is linked to various health benefits, such as reducing cancer risk and improving immune system functioning, in addition to its established benefit in musculoskeletal health ^12– 14^. Other mechanisms may also contribute to health. Evidence suggests that sunlight converts nitric oxide metabolites, stored in the skin, to nitric oxide ^15^, which may help to reduce blood pressure, amongst other actions that may be beneficial to cardiovascular health ^16^. Furthermore, sun protection advice often fails to consider different skin types fully. People of different skin types react to UVR in different ways, resulting in different needs. A recent review found little evidence of an association between melanoma incidence and UVR in people with skin of colour ^17^, suggesting that sunlight exposure may not be a risk factor for melanoma in those with darker skin, as it is for those with lighter skin ^18^.

Given the various risks and benefits associated with sunlight exposure, there is a need to examine the overall effect on population mortality to help people find the right balance between gaining the benefits of sunlight exposure whilst minimizing the risks. We sought to examine the global evidence on the association between sunlight exposure and mortality. Our main aims were to estimate the effect of exposure to sunlight on all-cause mortality, cardiovascular-related mortality and cancer-related mortality. We additionally examined evidence on whether the effects of exposure to sunlight on mortality vary according to skin type/colour or ethnicity. We undertook a systematic review following principles outlined in the Centre for Reviews and Dissemination (CRD) guidance for undertaking reviews in health care ^19^ and the *Cochrane Handbook for Systematic Reviews of Interventions* ^20^.

## Methods

### Patient and public involvement

We formed a public advisory group comprising four individuals with different skin types (three with Afro-Caribbean ancestry and one with very pale skin). We obtained input into the protocol from this group, although their ability to influence it was limited since the project was commissioned. They explained that people with darker skin can be very concerned about the harms of sunlight exposure as well as the possibility of vitamin D deficiency, reinforcing the need to examine data by subgroups defined by skin colour and type. They also made it clear that more refined guidance would be useful for people with all types of skin. They strongly supported the project and advised us that the findings would be of great interest. They further advised us on how we might disseminate the findings to those responsible for national guidelines on sun exposure; they felt that their health providers (e.g. general practitioners) were not sufficiently informed to provide targeted advice. Members of the public helped us write a lay summary of our plans for our website in advance of the project start ( https://bristol-esg.org/projects-2/sunlight-exposure/) and a member of the public advisory group helped us write a plain language summary to disseminate the results.

### Eligibility criteria and search methods

Criteria for inclusion of articles in the review were: (i) reporting a primary epidemiological study with a cohort design (including randomized trials of interventions to alter relevant behavioural exposures), a case-control design or an ecological design; (ii) in a general population; (iii) using any measure(s) of long-term sunlight exposure; and (iv) reporting outcome data on all-cause mortality, cancer-specific mortality or cardiovascular disease (CVD) mortality. To perform a comprehensive assessment, we used the exposure term ‘sunlight’ broadly to include measures of radiation (such as sunlight, UVR and UVB); proxies for radiation measures (such as latitude and geographical location); and behaviours associated with sun exposure (such as episodes of sunburn and recreational sun exposure). We excluded: (i) studies of artificial sources of UVR exposure, such as sunbed use and prescribed phototherapy and (ii) studies restricted to people with pre-existing disease (e.g. reporting survival rates in people diagnosed with melanoma).

We searched MEDLINE, Embase, Web of Science and the Cochrane Central Register of Controlled Trials to November 2023 using relevant controlled vocabularies, text‐words and search syntax appropriate to each resource. See Appendix S1 in the *extended data* ^21^ for the full search strategy. Additionally, we performed forwards and backwards citation searches of included articles identified in the search and scanned the reference lists of relevant systematic reviews. We also checked for any relevant retraction statements or errata of included studies. Two reviewers independently assessed all reports for eligibility.

### Data extraction and analysis

Data were extracted using standardized data extraction forms developed in Microsoft Excel, which had first been piloted on a small sample of articles and adapted as necessary. We collected the following data: study design (nested case-control, case-control, cohort, ecological, trial, quasi-experimental), funding sources (public, industry, mixed), study location, sex, age, ethnicity/race, skin type/colour, occupation, inclusion criteria, method/definition of sunlight exposure, period of exposure (childhood, adolescence, adulthood), length of exposure (specific, lifetime) and target condition (all-cause, cancer, CVD). We extracted summary data relating to the association between sun exposure and mortality overall and for CVD and cancer-related causes. This was reported differently across the studies included in this review, and included odds ratios (OR), risk ratios (RR), hazard ratios (HR), regression coefficients, correlation coefficients and narrative reporting.

Our main outcomes were death from any cause (all-cause mortality), death due to any cancer (all-cancer mortality) and death due to any cardiovascular cause ^22^ (all-CVD mortality). We also examined death due to skin cancer (melanoma and NMSC) and the five most common causes of cancer-related mortality in the UK according to Cancer Research UK ^23^: breast, prostate, lung, bowel and pancreatic cancer; as well as specific causes of CVD.

We assessed risk of bias in results using the Risk Of Bias In Non-randomized Studies of Exposure (ROBINS-E) tool for observational studies ^24^. Risk-of-bias assessments were not carried out on articles that reported results as correlations, mortality rates or narratively because the ROBINS-E tool aims to assess risk of bias in estimates of causal effects. Two reviewers independently assessed all reports considered potentially relevant for inclusion, extracted descriptive data and results of included articles, and assessed the risk of bias in results. Any disagreements were resolved by consensus or discussion with a third reviewer.

Due to the diversity in the types and units of measurement of the sunlight exposure measures, we did not consider it appropriate to conduct meta-analyses. Instead, we produced a narrative synthesis of the findings for each mortality outcome and examined any comparisons across people of different skin type/colour or ethnicity.

Articles reporting results as a relative risk (e.g., RR, HR; or data sufficient to derive these) were grouped by type of exposure and presented in forest plots, with estimates inverted as appropriate to illustrate findings using consistent direction of effect. Where an article reported only separate risk estimates for males and females, we combined these using fixed-effect meta-analysis to provide a result that additionally controlled for sex/gender.

Where results were presented in forms other than relative risks for two or more studies, we listed these in tables. Where such results were reported only separately for males and females these were treated as one analysis result (to be consistent with the combined relative risk results), but were reported as separate results in the tables.

We address certainty in the evidence through consideration of each of the five GRADE domains in the discussion.

## Results

### Search results and included studies

**Figure 1.**
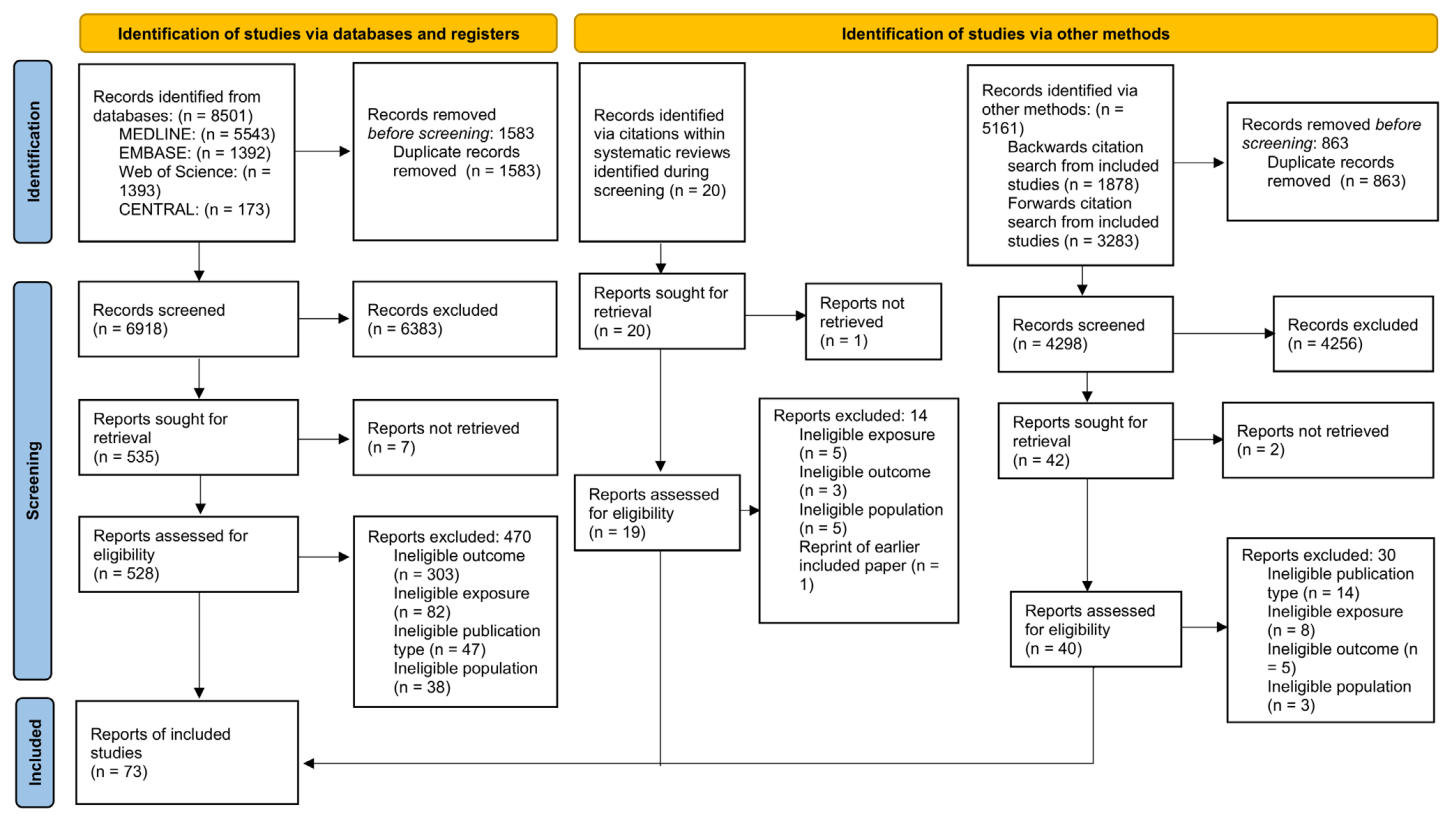
Identification of relevant studies.

The database searches identified 8501 records, and 5181 records were identified using other methods. After examination of potentially relevant full text articles, 73 articles ^25– 97^ met the criteria for inclusion in the review ( Figure 1). For a comprehensive summary of the characteristics of these 73 articles, see Table S1 in the *extended data* ^21^.

Where multiple articles had used the same cohort, same exposure type and reported the same outcome, we classed these as ‘overlapping’. In these cases, we chose a main article as the source of data. The selection of data was based on an algorithm that included the type of analysis performed (ratio data preferred to linear regression or correlation coefficients), adjustment within analysis (most relevant confounders controlled for preferred), population (whole population preferred to specific subgroups), follow-up time (longer preferred), number of units of observation (e.g. counties preferred to states) and number of participants. As the majority of the included studies were ecological, we applied the same process to national datasets. We considered articles using national mortality data for the same country and date range to be using the same cohort, and used the same selection algorithm to select a main article in such cases. Table S2 in the *extended data* ^21^ provides a review of all overlapping studies.

After the selection of main articles, this resulted in 55 articles being included in the narrative synthesis. All subsequent analysis is conducted on these 55 articles. Eight of the articles reported data on all-cause mortality, eight on all-CVD mortality, and 17 on all-cancer mortality. Eleven described cohort studies, three case-control studies and 41 ecological studies.

Twenty-four (44%) of the articles were conducted in the USA, five were conducted worldwide (including between 34 and 172 countries), four in China, and three each in Japan, Spain and Sweden. Other articles were conducted in the USA and Canada, the UK, and Switzerland (all n = 2); as well as Australia, Chile, Europe-wide, France, Italy, New Zealand and Turkey (all n = 1).

We provide descriptions of the exposures in Table S3 in the *extended data* ^21^. The majority of the articles (n = 42, 76%) measured a single exposure; ten (18%) measured two exposures, two (4%) measured three exposures and one measured four exposures. Thus, we reported details of 72 exposures in total. Of those, 40 were measures of radiation, 17 were proxy measures of radiation and 15 were measures of behaviours associated with sunlight exposure. Radiation measures included solar radiation, UVR, UVA and UVB, DNA- and erythemal-weighted UVB and UVR, UV Index, solar incidence, insolation, irradiation, sunlight hours and sunshine. Among the proxy measures for radiation, 16 were latitude (e.g. residential at county or state level) and one was geographic location of deployment during war (tropical *vs* European). Measures of sunlight exposure-related behaviour included four of occupational exposure, four of recreational exposure and one of both occupational and recreational exposure combined; three used skin damage, two used NMSC mortality rates and one used melanoma mortality rates.

Of the 55 included main articles, 34 (62%) reported information on participants’ skin type/colour or ethnicity. Most were conducted in only White participants, whilst two only contributed data for Black participants. In six articles, the authors reported that skin type/colour or ethnicity was mixed. Of these, four included Black and White participants (although, where proportions were reported, the populations were predominately White). One included Black, White and Hispanic participants, and one included Black, White, Hispanic, Asian and Native American participants.

Below we provide our findings for our primary outcomes: all-cause, all-CVD and all-cancer mortality, both overall and by type of exposure. Subsequently, we provide a brief summary of the findings for our secondary, cause-specific mortality outcomes. We report the full risk-of-bias assessments, including justifications for judgments, in Table S4 in the *extended data* ^21^.

### All-cause mortality

**Figure 2.**
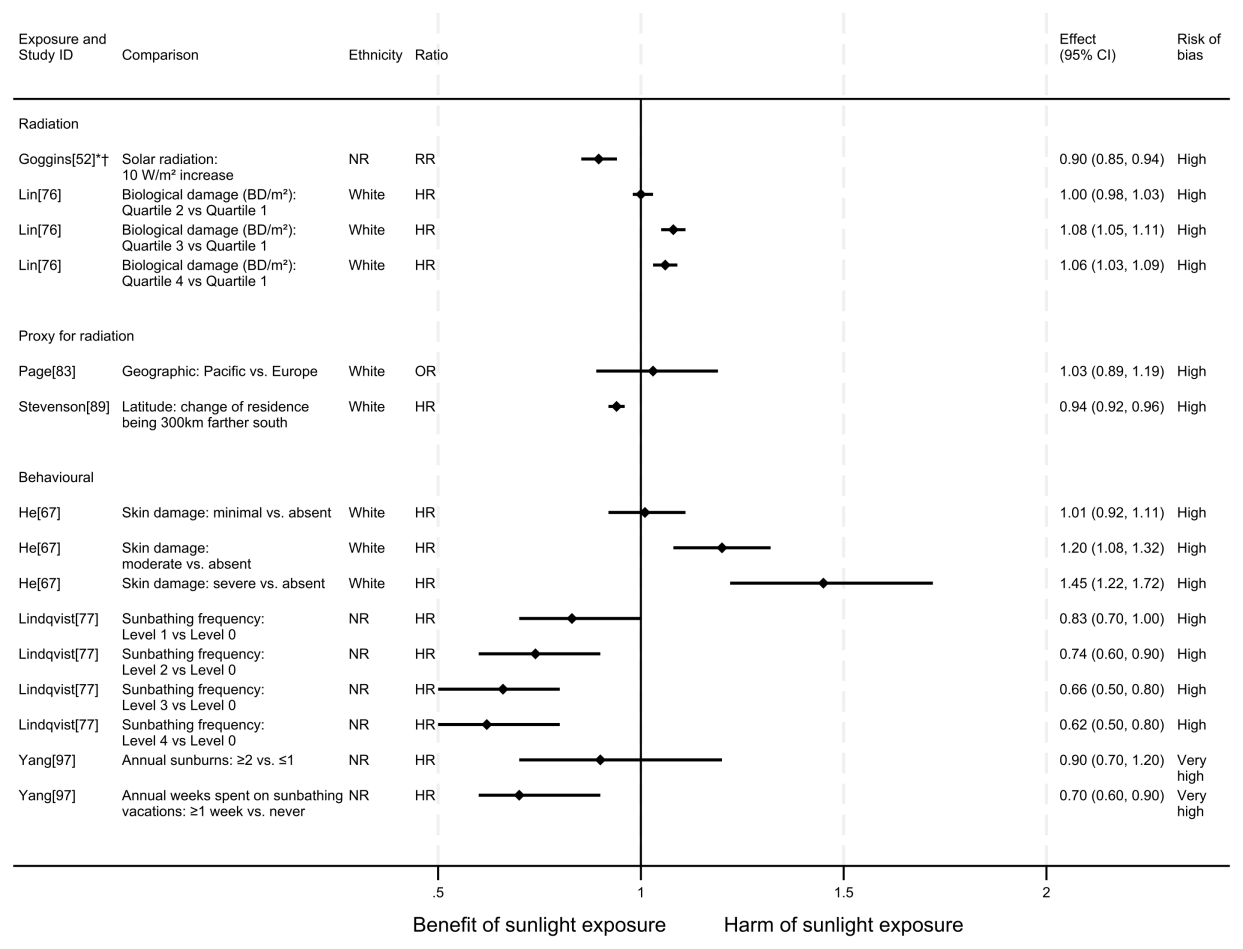
Forest plot of studies showing the effect of exposure on all-cause mortality. *Fixed-effects meta-analysis performed to combine gender subgroups. †Result inverted to reflect an increase in exposure. NR: ethnicity of population not reported.

***Overview.*** In total, eight articles investigated the effect of sunlight on all-cause mortality. Six used a cohort design and two had an ecological design. One article reported findings for two exposures (nine analyses reported across the eight articles). The findings were mixed (see Figure 2 for reported relative risks). Five analysis results ^52, 77, 89, 97^ were in the direction of a beneficial association between sunlight and all-cause mortality. Four analysis results ^47, 67, 76, 83^ were in the direction of a harmful effect of sunlight.

**Figure 3.**
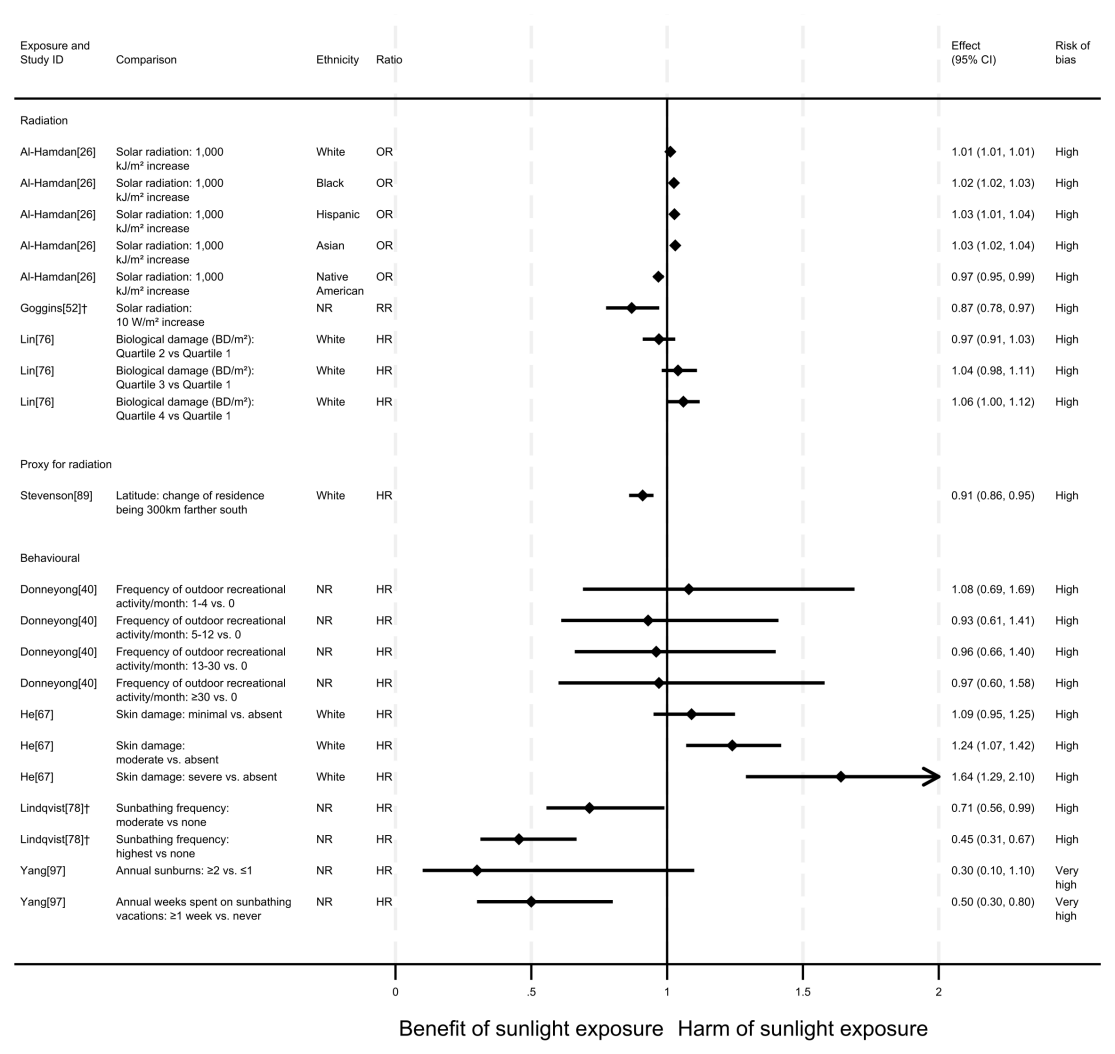
Forest plot of studies showing the effect of exposure on all-CVD mortality. †Result inverted in order to reflect an increase in exposure. NR: ethnicity of population not reported.

***Radiation.*** Three articles looked at the effect of radiation on all-cause mortality ( Figure 2). The results of all three articles were considered to be at high risk of bias. Goggins *et al.* ^52^ conducted an ecological study of the Hong Kong population showing that a 10 W/m ^2^ increase in solar radiation was associated with a 10% decrease in all-cause mortality (RR = 0.90, 95% confidence interval (CI) 0.85 to 0.94). Lin *et al.* ^76^ measured the residential address of cohort participants across six states and two metropolitan areas in the USA to determine average July erythemal UVR, defined as biological damage per square meter (BD/m ^2^). This measure was subsequently split into quartiles. When comparing the second quartile with the first, there was no evidence of a difference in all-cause mortality (RR = 1.00, 95% CI 0.98 to 1.03; Figure 3). However, when comparing the third and fourth quartiles with the first there were 8% and 6% increases in all-cause mortality, respectively.

In addition to the articles included in Figure 2, Fu and Wang ^47^ examined the relationship between the average hours of daily sunshine in China and all-cause mortality using regression. They found that an increase of 0.1 to 0.2 in the average daily sunshine duration rate (equivalent to 2.9 hours of additional daily sunshine) was associated with an increase in all-cause mortality rate the following year (β = 11.509, 95% CI 1.87 to 21.15; high risk of bias).

***Proxy for radiation.*** Two articles looked at the effect of proxy radiation exposures on all-cause mortality ( Figure 2). The results of both articles were considered to be at high risk of bias. Stevenson *et al.* ^89^ examined the effect of latitude in the UK. They found that more southerly latitudes were associated with lower all-cause mortality, when compared with residences 300km further north (HR = 0.94, 95% CI 0.92 to 0.96). Page *et al.* ^83^ examined a cohort of WWII veterans who were deployed to either Pacific or European battlefronts, arguing that those who were deployed to the Pacific area would have experienced higher levels of sun exposure than those in Europe. They observed a small increase in risk associated with being deployed in the Pacific compared with Europe, however the wide confidence intervals were compatible with both a benefit and harm (odds ratio = 1.03, 95% CI 0.89 to 1.19).

***Behavioural.*** Three articles examined the effect of sunlight exposure behaviour ( Figure 2). He *et al.* ^67^ looked at the effect of physician-assessed actinic skin damage. The findings suggested that greater sun exposure, as indicated by skin damage, is associated with an increase in the risk of mortality. When comparing those with severe skin damage with those who were assessed to have no damage, there was a 45% increase in risk (HR = 1.45, 95% CI 1.22 to 1.72; high risk of bias). The association does not appear to be driven by smoking (an important common cause of skin damage and CVD), which was controlled for in the analysis.

Lindqvist *et al.* ^77^ measured self-reported sun exposure in a cohort of Swedish women. The evidence suggested that there may be a decrease in mortality with increased sun exposure. Those who reported the highest level of sun seeking behaviour had a 38% decreased risk of dying from any cause compared with those who reported the lowest level (HR = 0.62, 95% CI 0.50 to 0.80; high risk of bias). This finding is supported by Yang *et al.* ^97^ in another cohort of Swedish women, though their results were considered to be at very high risk of bias. Those who reported spending one week or more annually on sunbathing vacations had a 30% decreased risk of mortality compared with those who never went on sunbathing vacations (HR = 0.70, 95% CI 0.60 to 0.90). They also reported an association between higher number of sunburns experienced and lower all-cause mortality, however the wide confidence intervals were compatible with both a benefit and harm (HR = 0.90, 95% CI 0.70 to 1.20).

### All-CVD mortality

***Overview.*** In total, eight articles investigated the effect of sunlight on overall CVD mortality, with one reporting findings for two exposure types (thirteen analyses reported across the eight articles). Six of the articles had a cohort design and two were ecological. There were mixed findings ( Figure 3). Six analysis results ^26, 52, 78, 89, 97^ suggested that higher levels of sunlight are associated with lower risk of CVD mortality. In contrast, five analyses ^26, 67^ suggested that higher levels of sunlight are associated with a higher risk of CVD mortality. Two analyses ^40, 76^ produced mixed findings.

***Radiation.*** Three articles looked at the effect of radiation, all were considered to be at high risk of bias ( Figure 3). Al-Hamdan *et al.* ^26^ performed a national ecological study in the USA. They found that a 100 kJ/m ^2^ increase in solar radiation was associated with a 1% higher risk of dying from CVD for White participants, a 3% higher risk for Black, Hispanic and Asian participants, but a 3% lower risk for Native American participants. Goggins *et al.*’s ^52^ Hong Kong-based study found that an increase in solar radiation of 10 W/m ^2^ was associated with a lower risk of dying from CVD (RR = 0.87, 95% CI 0.78 to 0.97). In Lin *et al.*’s ^76^ dose response analysis of average July residential erythemal UVR exposure (BD/m ^2^), there were mixed findings. Risk of CVD mortality increased with dose. However, across all levels of comparison, the confidence intervals were compatible with both a benefit and harm, or with a null effect.

***Proxy for radiation.*** Stevenson *et al.* ^89^ found evidence to suggest a beneficial effect of sunlight exposure, measured via latitude in the UK. Compared with northerly latitudes, residences 300 km further south were associated with a 9% lower risk of CVD mortality (HR = 0.91, 95% CI 0.86 to 0.95; high risk of bias).

***Behavioural.*** Four articles examined the effect of sunlight exposure behaviour ( Figure 3). Lindqvist’s *et al.* ^78^ Swedish cohort study found that those who had self-reported high recreational sun exposure were less at risk of dying from CVD than those who reported no exposure (HR = 0.45, 95% CI 0.31 to 0.67; high risk of bias).

Yang *et al.* ^97^ found that people who reported spending one or more weeks a year on sunbathing vacations were half as likely to die from CVD than those who never spent time on sunbathing vacations (HR = 0.50, 95% CI 0.30 to 0.80). Additionally, there was an association between higher frequency of sunburning and lower CVD mortality, however the wide confidence intervals indicate uncertainty in this finding (HR = 0.30, 95% CI 0.10 to 1.10). Furthermore, the results reported in this article were considered to be at very high risk of bias.

In contrast, the findings in He *et al.* ^67^ suggested a harmful effect of sunlight. They found that greater physician-assessed actinic skin damage was associated with a higher risk of CVD mortality. Those whose skin damage was considered severe had a 64% increased risk of CVD mortality compared with those with no actinic skin damage (HR = 1.64, 95% CI 1.29 to 2.10; high risk of bias). As shown for all-cause mortality, the association does not appear to be driven by smoking, which was controlled for in the analysis.

Donneyong *et al.* ^40^ looked at the self-reported frequency of outdoor recreational activity. The results were mixed, with wide confidence intervals that were compatible with both a benefit and harm, and considered to be at high risk of bias.

### All-cancer mortality

**Figure 4.**
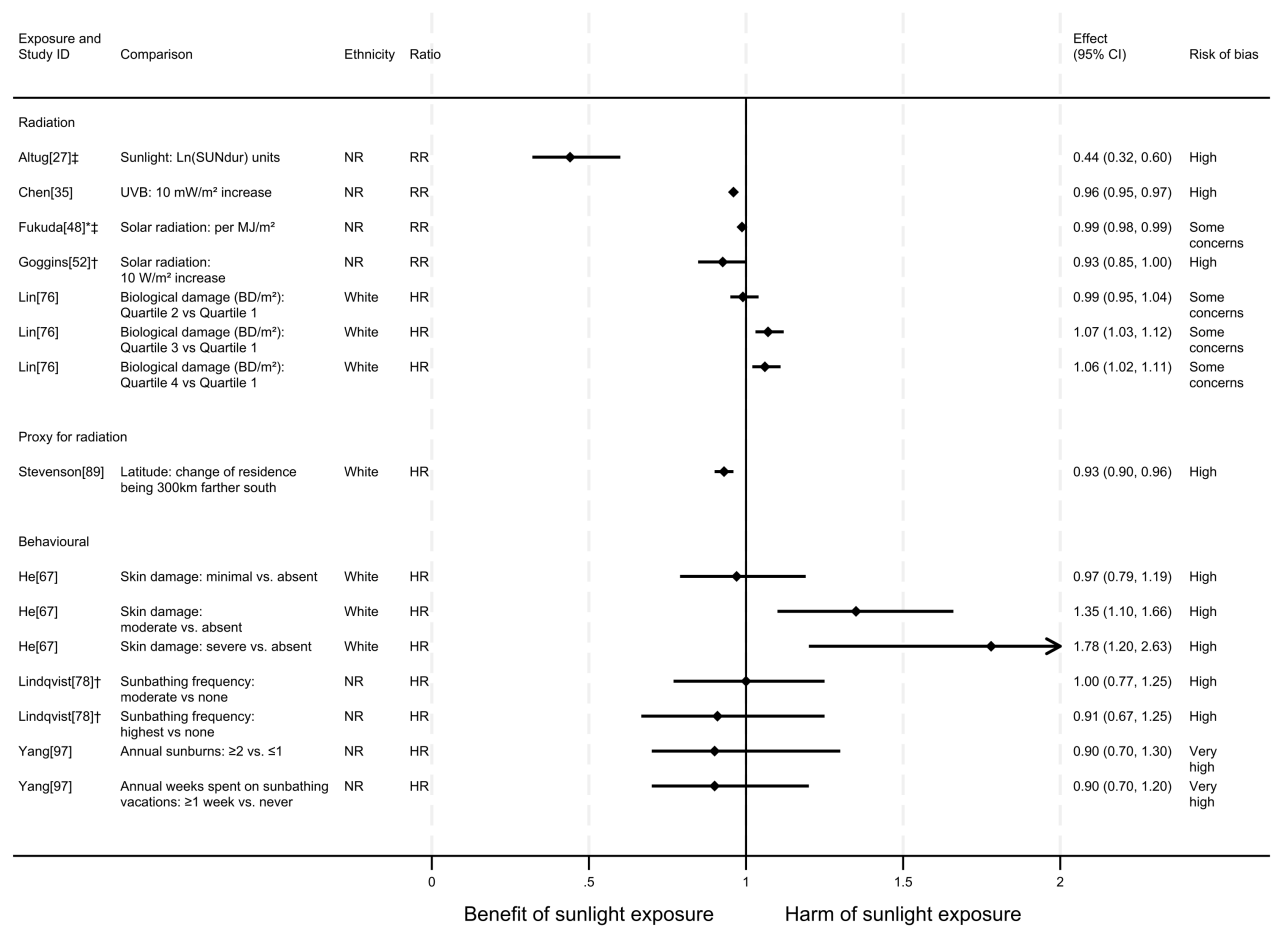
Forest plot of studies showing the effect of exposure on all-cancer mortality. *Fixed-effects meta-analysis performed to combine gender subgroups. †Result inverted in order to reflect an increase in exposure. ‡Ratio calculated via exponentiating logistic regression beta coefficient. NR: ethnicity of population not reported.

***Overview.*** In total, 17 articles looked at the effect of sunlight exposure on all-cancer mortality, with some reporting multiple exposure types, outcomes and/or date ranges (22 analyses reported across the 17 articles). Twelve of the articles had an ecological design and five had a cohort design. The majority of analysis results (20 analyses) were in the direction of a beneficial association between sunlight and all-cancer mortality. However, two analyses ^67, 76^ suggested there may be a harmful effect of sunlight. See Figure 4 for reported relative risks and Table 1 for results reported in other formats.

**Table 1.** Linear regression, correlation and narrative results showing the effect of exposure on all-cancer mortality.

Study	Location	Exposure	Unit of analysis	Specific Outcome	Subgroup	Analysis	Results	Direction of effect	Risk of bias
Apperly ^28^	USA and Canada	Radiation (solar radiation)	Solar radiation Index	All-cancer	n/a	Correlation	r = −0.63	Benefit	n/a
		Behavioural (occupational exposure to sunlight)	% of farmers per state population	All-cancer	n/a	Correlation	r = −0.68	Benefit	n/a
Camara and Brandao ^34^	Worldwide	Radiation (solar incidence)	kWh/m ^2^ / day	All-cancer	n/a	Mortality rate	“92.48/100,000 in countries with high sunlight incidence 124.85/100,000 in countries with low sunlight incidence” (p < 0.05)	Benefit	n/a
Ezzati *et al.* ^42^	USA	Radiation (insolation)	Annual average solar radiation	All-cancer (smoking- related)	Males	Regression	β = −0.00029 (95% CI −0.00054 to −0.000031)	Benefit	Some concerns
					Females	Regression	β = −0.00033 (95% CI −0.00051 to −0.00015)	Benefit	Some concerns
				All-cancer (non- smoking related	Males	Regression	β = −0.00032 (95% CI −0.00057 to −0.000064)	Benefit	Some concerns
					Females	Regression	β = −0.00079 (95% CI −0.001 to −0.00055)	Benefit	Some concerns
Grant and Garland ^56^ (1950–1969)	USA	Radiation (UVB)	kJ/m ^2^	All- cancer **	White males	Regression	β = −0.65 (p < 0.001)	Benefit	Some concerns
					White females	Regression	β = −0.85 (p < 0.001)	Benefit	Some concerns
Grant and Garland ^56^ (1970–1994)	USA	Radiation (UVB)	kJ/m ^2^	All- cancer **	White males	Regression	β = −0.54 (p < 0.001)	Benefit	Some concerns
					White females	Regression	β = −0.82 (p < 0.001)	Benefit	Some concerns
Grant ^57^	USA	Radiation (UVB)	kJ/m ^2^	All- cancer **	Black males	Regression	β = −0.34 (p = 0.01)	Benefit	Very high
					Black females	Regression	β = −0.50 (p < 0.001)	Benefit	Very high
Grant ^59^	China	Proxy for radiation (latitude)	Degree of latitude *	All-cancer	Females	Regression	β = 0.75 (p < 0.001)	Benefit	High
Grant ^62^	France	Proxy for radiation (latitude)	Degree of latitude squared *	All-cancer	Male	Correlation	r = 0.8 (p < 0.001)	Benefit	High
					Female	Correlation	r = 0.78 (p < 0.001)	Benefit	High
Grant ^64^	USA (California)	Proxy for radiation (latitude)	Degree of latitude *	All-cancer	n/a	Regression	β = 0.47 (p = 0.009)	Benefit	High
		Behavioural (NMSC mortality)	Mortality rate	All-cancer	n/a	Regression	β = −0.69 (p < 0.001)	Benefit	High

Note. *An increase in latitude indicates a decrease in sunlight exposure. Therefore, a positive relationship between latitude and mortality suggests a protective effect of sunlight. **not including lung cancer. Abbreviations: b: regression beta coefficient; CI: confidence interval; n/a: not applicable; NMSC: non-melanoma skin cancer; r: correlation coefficient; RoB: risk of bias; UVB: ultraviolet B radiation.

***Radiation.*** Ten articles looked at the effect of radiation on all-cancer mortality. The majority (n = 9) found a potentially beneficial effect of sunlight, with higher levels of radiation associated with lower levels of cancer mortality ( Figure 4 and Table 1). In Altug and Kilçiksiz ^27^, a national ecological study in Turkey, the results indicated that a one (unspecified) unit increase in sunlight duration was associated with a 56% reduction in the risk of cancer mortality (RR = 0.44, 95% CI 0.32 to 0.60; high risk of bias).

Chen *et al.* ^35^ performed a national ecological study in China, looking at the effect of UVB exposure on cancer mortality. The findings suggested that a 10 mW/m ^2^ increase in average daily UVB irradiance was associated with a 4% lower risk of mortality (rate ratio = 0.96, 95% CI 0.95 to 0.97; high risk of bias). Similarly, Fukuda *et al.*’s ^48^ ecological study in Japan found that, per MJ/m ^2^ of solar radiation, there was a 1.3% reduction in cancer mortality risk (RR = 0.99, 95% CI 0.98 to 0.99; some concerns over risk of bias). The findings in Grant and Garland ^56^ and Grant ^57^ suggest a similar association. Higher levels of UVB were found to be associated with lower cancer mortality rates across both Black and White males and females between 1970 and 1994 (some concerns over risk of bias). A relationship between higher levels of radiation and lower cancer mortality was also observed in Apperly ^28^, Camara and Brandao ^34^, Ezzati *et al.* ^42^ (some concerns over risk of bias) and Grant and Garland ^56^ (1950–1969; some concerns over risk of bias).

Goggins *et al.* ^52^ found an association between higher solar radiation and lower cancer mortality, however the confidence intervals were compatible with a null effect (RR = 0.93, 95% CI 0.85 to 1; high risk of bias). In contrast, Lin *et al.* ^76^ found that increasing levels of residential erythemal UVR (BD/m ^2^) were associated with an increased risk of cancer mortality. Comparing the fourth with the first quartile showed a 6% increase in the risk of mortality (HR = 1.06, 95% CI 1.02 to 1.11; some concerns over risk of bias).

***Proxy for radiation.*** Four articles looked at the effect of proxy radiation exposures on cancer mortality, with all finding a potentially beneficial effect of sunlight ( Figure 4 and Table 1). The findings of all four of the articles were assessed to be at high risk of bias.

Stevenson *et al.* ^89^ reported that, compared with UK northerly latitudes, residences 300 km further south had a 7% reduction in mortality risk (HR = 0.93, 95% CI 0.90 to 0.96). Grant ^59^ conducted a national ecological study in China. They found an association between higher latitudes (i.e., lower levels of sunlight) and higher cancer mortality (β = 0.75, p < 0.001). A similar relationship was found by Grant in France ^62^ and California ^64^.

***Behavioural.*** Five articles studied the effect of sunlight exposure behaviour on cancer mortality ( Figure 4 and Table 1). He *et al.* ^67^ found that those with greater physician-assessed actinic skin damage had a greater risk of cancer mortality. Participants whose skin damage was considered severe had a 78% higher risk of mortality compared with those with no actinic skin damage (HR = 1.78, 95% CI 1.20 to 2.63; high risk of bias).

In contrast, Grant’s ^64^ ecological study in California, using population-level NMSC mortality rates as a proxy for sunlight exposure, found a relationship between higher NMSC mortality rates and lower overall cancer mortality (β = −0.69, p < 0.001; high risk of bias). Apperly ^28^ looked at the proportion of the population engaged in agricultural work and reported a relationship between higher proportions of agricultural workers and lower cancer mortality. However, no margins of error were reported with this estimate.

Two articles, Lindqvist *et al.* ^78^ and Yang *et al.* ^97^, found that recreational sunbathing behaviour was associated with lower cancer mortality. However, in both cases there were wide confidence intervals which were compatible with both a beneficial and harmful effect of sunlight exposure ( Figure 4). Furthermore, they were considered to be at high and very high risk of bias, respectively.

### Specific cancers and CVD causes

***Skin cancers.*** In total, 23 articles investigated the effect of sunlight on skin cancer mortality with some reporting multiple exposure types, outcomes or date ranges (46 analyses reported across the 23 articles; 28 analyses measuring effect on melanoma, 16 measuring effect on NMSC, and two measuring effect on both combined). Seventeen of the articles were ecological, three had a cohort design and three used a case-control design. There were 25 analyses looking at the effect of radiation, 16 on the effect of proxy radiation exposures and five looked at the effect of sunlight exposure behaviour.

**Figure 5.**
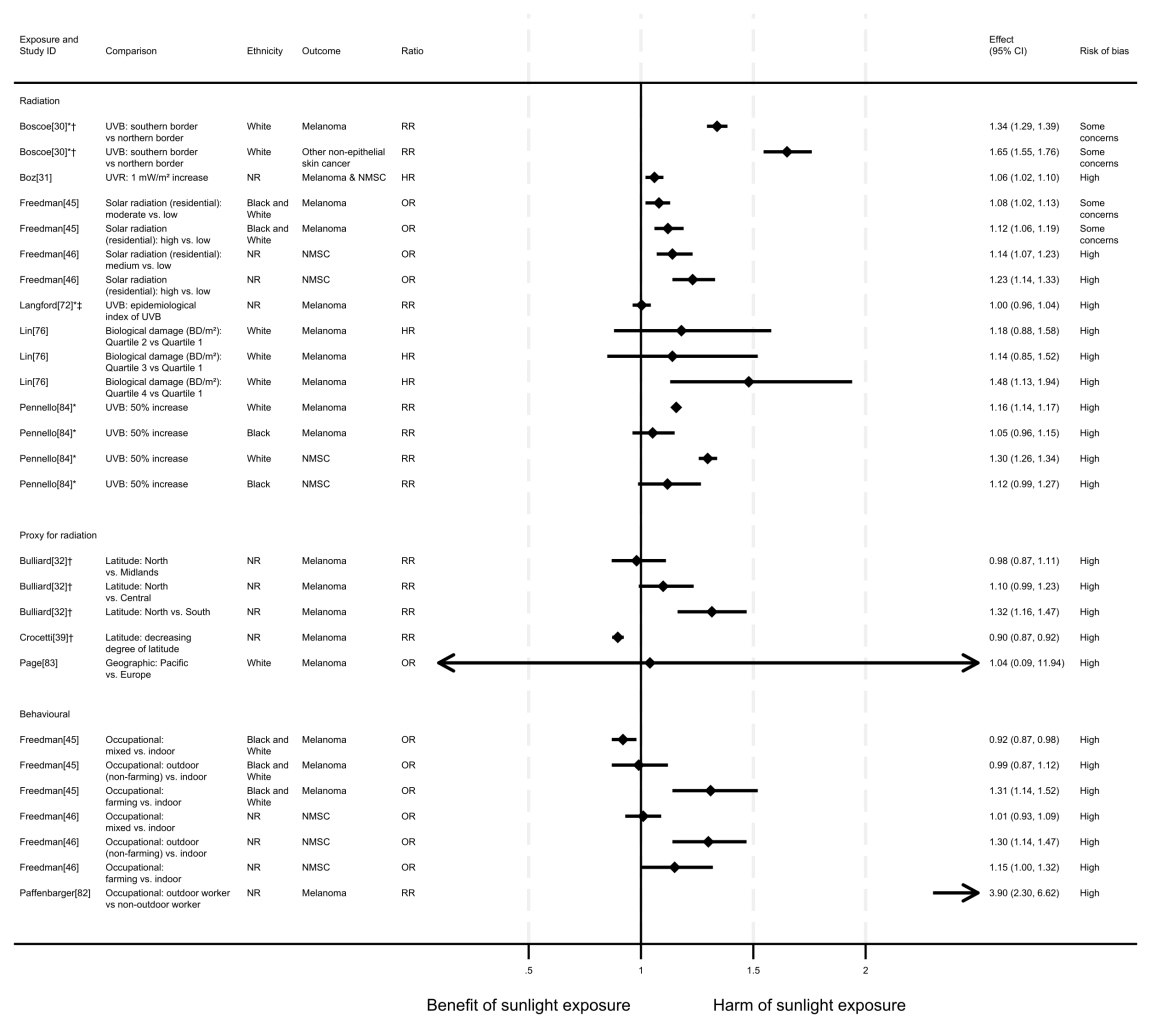
Forest plot of studies showing the effect of exposure on skin cancer mortality. *Fixed-effects meta-analysis performed to combine gender subgroups. †Result inverted in order to reflect an increase in exposure. ‡Ratio calculated via exponentiating logistic regression beta coefficient. NR: ethnicity of population not reported.

Most analysis results (n=34) suggested that higher levels of sunlight were associated with a higher risk of both melanoma and NMSC mortality ( Figure 5 and Table 2). However, the results of six were in the direction of a beneficial effect of sunlight: two found that higher latitude (i.e. lower sunlight) was associated with lower melanoma ^39^ and NMSC mortality ^25^; two suggested an association between higher levels of UVB and lower melanoma mortality ^51, 90^; one reported a weak association between higher annual insolation and lower melanoma mortality ^25^ and one reported an association between higher NMSC mortality rates and lower melanoma mortality ^58^. The remaining six analyses either produced mixed results ^45, 58, 96^, had no direction reported ^44^, showed little to no effect ^72^ or had very wide confidence intervals that were compatible with both a benefit and harm ^83^. 

**Table 2.** Linear regression, correlation and narrative results showing the effect of exposure on skin cancer mortality.

Study	Location	Exposure	Unit of analysis	Specific Outcome	Subgroup	Analysis	Results	Direction of effect	Risk of bias
Alcalá Ramírez del Puerto *et al.* ^25^	Spain	Radiation (irradiation)	NR	Melanoma	n/a	Correlation	r = 0.16 (p > 0.05)	Harm	n/a
				NMSC	n/a	Correlation	r = 0.40 (p > 0.05)	Harm	n/a
		Radiation (insolation)	NR	Melanoma	n/a	Correlation	r = −0.113 (p > 0.05)	Benefit	n/a
				NMSC	n/a	Correlation	r = 0.266 (p > 0.05)	Harm	n/a
		Proxy for radiation (latitude)	Degree of latitude *	Melanoma	n/a	Correlation	r = 0.22 (p > 0.05)	Harm	n/a
				NMSC	n/a	Correlation	r = −0.40 (p < 0.01)	Benefit	n/a
Elwood *et al.* ^41^	USA & Canada	Radiation (Epidemiological index of UVR)	NR	Melanoma	Males	Regression	β = 0.039 (p < 0.001)	Harm	High
					Females	Regression	β = 0.022 (p < 0.001)	Harm	High
				NMSC	Males	Regression	β = 0.045 (p < 0.001)	Harm	High
					Females	Regression	β = 0.017 (p < 0.001)	Harm	High
		Proxy for radiation (latitude)	Degree of latitude *	All skin cancer	Males	Regression	β = −0.12 (SE = 0.011)	Harm	High
					Females	Regression	β = −0.055 (SE = 0.009)	Harm	High
Fleischer and Fleischer ^44^	USA	Radiation (solar radiation)	kJ/m ^2^	Melanoma	n/a	Narrative	“No associations were demonstrated between solar energy and cancer mortality for melanoma of the skin (p = 0.60)”	NR	n/a
Garland *et al.* ^51^	Worldwide	Radiation (UVA)	1 photon flux per nanometer	Melanoma	Males	Regression	β = 0.00012 (SE = 0.000050)	Harm	High
					Females	Regression	β = 0.00004 (SE = 0.00003)	Harm	High
		Radiation (UVB)	1 photon flux per nanometer	Melanoma	Males	Regression	β = −0.00146 (SE = 0.00094)	Benefit	High
					Females	Regression	β = −0.00106 (SE = 0.00053)	Benefit	High
Grant ^58^	Spain	Proxy for radiation (latitude)	Degree of latitude *	Melanoma	Males	Correlation	r = −0.08 (p > 0.05)	Mixed (gender)	n/a
					Females	Correlation	r = 0.28 (p > 0.05)	Mixed (gender)	n/a
				NMSC	Males	Correlation	r = −0.5 (p < 0.01)	Harm	n/a
					Females	Correlation	r = −0.33 (p < 0.05)	Harm	n/a
		Behavioural (NMSC mortality)	Mortality rate	Melanoma	Males	Correlation	r = −0.03 (p > 0.05)	Benefit	n/a
					Females	Correlation	r = −0.43 (p < 0.01)	Benefit	n/a
Grant ^61^ (1950–1969)	USA	Proxy for radiation (latitude)	Degree of latitude *	NMSC	White males	Regression	β = −0.66 (p < 0.001)	Harm	High
					White females	Regression	β = −0.41 (p = 0.002)	Harm	High
Grant ^61^ (1970–1994)	USA	Proxy for radiation (latitude)	Degree of latitude *	NMSC	White males	Regression	β = −0.37 (p = 0.001)	Harm	High
Grant ^63^ (1950–1969)	USA	Radiation (UVB)	kJ/m ^2^	Melanoma	White males	Regression	β = 0.47 (p < 0.001)	Harm	High
					White females	Regression	β = 0.46 (p < 0.001)	Harm	High
				NMSC	White males	Regression	β = 0.34 (p < 0.001)	Harm	High
					White females	Regression	β = 0.21 (p < 0.001)	Harm	High
Grant ^64^	USA (California)	Behavioural (NMSC mortality)	Mortality rate	Melanoma	White males	Regression	β = 0.46 (p = 0.03)	Harm	High
Lee ^74^ (1950–1959)	USA	Proxy for radiation (latitude)	Degree of latitude *	Melanoma	White males	Regression	β = −0.039 (95% CI −0.050 to −0.028)	Harm	High
					White females	Regression	β = −0.037 (95% CI −0.053 to −0.021)	Harm	High
Lee ^74^ (1960–1969)	USA	Proxy for radiation (latitude)	Degree of latitude *	Melanoma	White males	Regression	β = −0.037 (95% CI −0.047 to −0.028)	Harm	High
					White females	Regression	β = −0.030 (95% CI −0.039 to −0.021)	Harm	High
Lee ^74^ (1970–1979)	USA	Proxy for radiation (latitude)	Degree of latitude *	Melanoma	White males	Regression	β = −0.026 (95% CI −0.036 to −0.016)	Harm	High
					White females	Regression	β = −0.023 (95% CI −0.032 to −0.013)	Harm	High
Lee ^74^ (1988–1992)	USA	Proxy for radiation (latitude)	Degree of latitude *	Melanoma	White males	Regression	β = −0.014 (95% CI −0.023 to −0.005)	Harm	High
					White females	Regression	β = −0.009 (95% CI −0.019 to −0.002)	Harm	High
Rivas *et al.* ^85^	Chile	Proxy for radiation (latitude)	Degree of latitude *	Melanoma	n/a	Correlation	r = −0.88	Harm	n/a
				NMSC	n/a	Correlation	r = −0.53	Harm	n/a
Takahashi *et al.* ^90^	Japan	Radiation (UVB)	10 J/m ^2^ /year	Melanoma	Males	Correlation	r = −0.155 (p = 0.35)	Benefit	n/a
					Females	Correlation	r = −0.09 (p = 0.58)	Benefit	n/a
				NMSC	Males	Correlation	r = 0.268 (p = 0.09)	Harm	n/a
					Females	Correlation	r = 0.282 (p = 0.08)	Harm	n/a
Wu and Weinstock ^96^	USA	Radiation (UV index)	UV index zone 2 *vs* UV index zone 1	Keratinocyte carcinoma	White males	Narrative	“White male KC mortality rate was found to be higher in sun Zone 2 (p = 0.004)”	Mixed (gender)	n/a
					White females	Narrative	“There was no statistical difference between sun zones for White women (p = 0.379)”	Mixed (gender)	n/a

Note. *An increase in latitude indicates a decrease in sunlight exposure. Therefore, a positive relationship between latitude and mortality suggests a protective effect of sunlight. Abbreviations: b: regression beta coefficient; CI: confidence interval; n/a: not applicable; KC: keratinocyte carcinoma; NMSC: non-melanoma skin cancer; NR: not reported; r: correlation coefficient; RoB: risk of bias; SE: standard error; UV: ultraviolet radiation. UVA: ultraviolet A radiation; UVB: ultraviolet B radiation.

**Figure 6.**
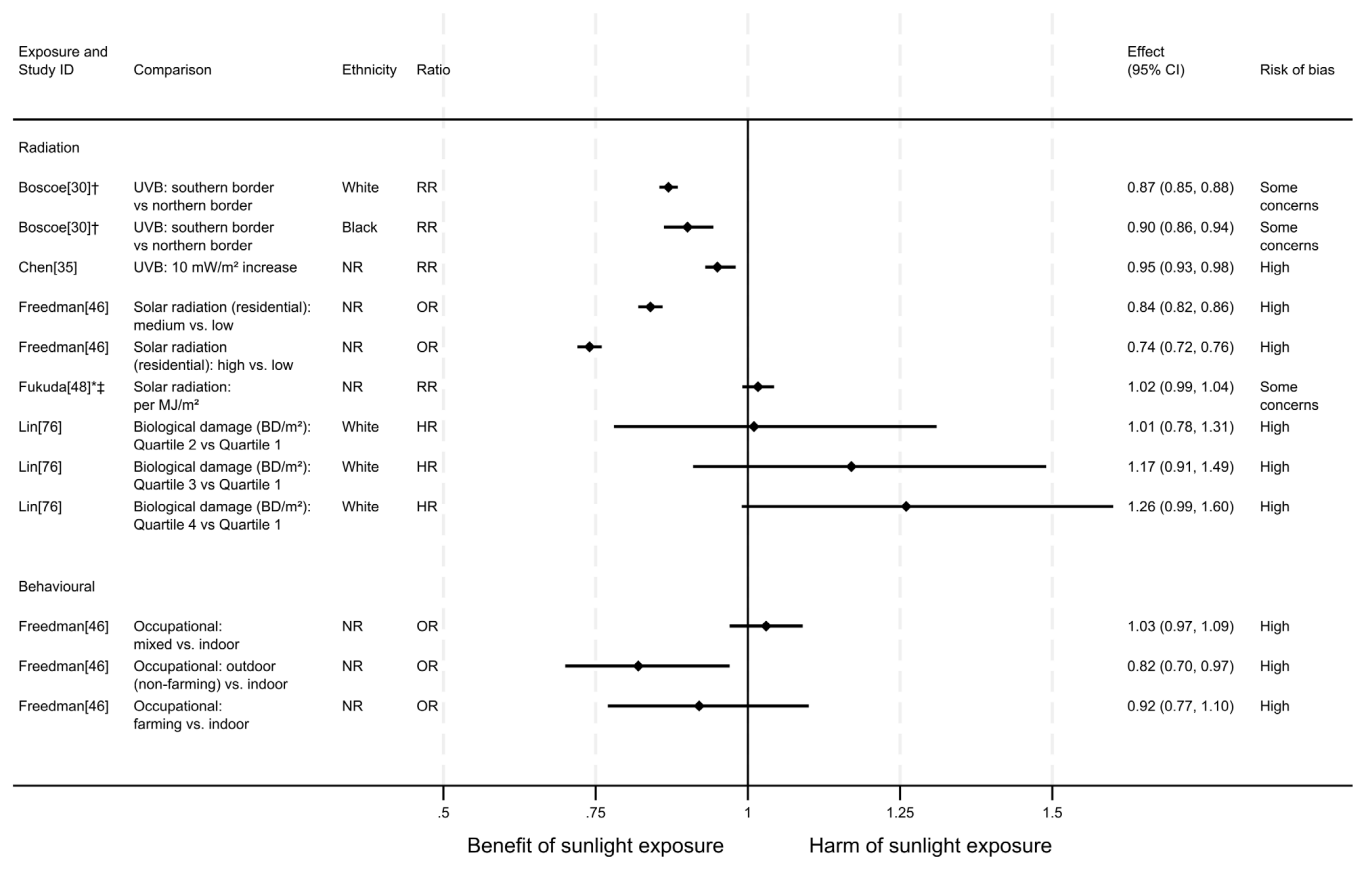
Forest plot of studies showing the effect of exposure on breast cancer mortality. *Fixed-effects meta-analysis performed to combine gender subgroups. †Result inverted in order to reflect an increase in exposure. ‡Ratio calculated via exponentiating logistic regression beta coefficient. NR: ethnicity of population not reported.

***Breast cancer mortality.*** In total, 11 articles looked at the effect of sunlight on breast cancer mortality, with some reporting multiple exposure types and/or date ranges (17 analyses reported, across the 11 articles). Nine articles had an ecological design and one each used cohort and case-control designs. There were ten analysis results examining the effect of radiation, four looked at proxy measures of radiation and three looked at behavioural measures of exposure. Overall, the evidence suggested that sunlight may provide a protective effect ( Figure 6 and Table 3). Thirteen analysis results from Boscoe and Schymura ^30^, Chen *et al.* ^35^, Freedman *et al.* ^46^, Grant and Garland ^56^, and Grant ^53, 57, 58, 62^ indicated that higher levels of sunlight were associated with reduced breast cancer mortality. Three analyses from Lin *et al.* ^76^, Fukuda *et al.* ^48^, and Grant ^58^ suggested that there may be a harmful association between sunlight and breast cancer mortality, and one from Fleischer and Fleischer ^44^ was reported without a direction of effect.

**Table 3.** Linear regression, correlation and narrative results showing the effect of exposure on breast cancer mortality.

Study	Location	Exposure	Unit of analysis	Specific Outcome	Subgroup	Analysis	Results	Direction of effect	Risk of bias
Fleischer and Fleischer ^44^	USA	Radiation(solar radiation)	kJ/m ^2^	Breast cancer	n/a	Narrative	“No associations were demonstrated between solar energy and cancer mortality for breast cancer (p = 0.40)”	Not reported	n/a
Grant ^53^	Worldwide	Proxy for radiation (latitude)	Degree of latitude *	Breast cancer	n/a	Correlation	r = 0.66 (p < 0.001)	Benefit	n/a
Grant and Garland ^56^ (1950–1969)	USA	Radiation (UVB)	kJ/m ^2^	Breast cancer	White females	Regression	β = −0.59 (p < 0.001)	Benefit	Some concerns
Grant and Garland ^56^ (1970–1994)	USA	Radiation (UVB)	kJ/m ^2^	Breast cancer	White males	Regression	β = −0.71 (p = 0.006)	Benefit	Some concerns
					White females	Regression	β = −0.71 (p < 0.001)	Benefit	Some concerns
Grant ^57^	USA	Radiation (UVB DNA)	kJ/m ^2^	Breast cancer	Black females	Regression	Β = −0.38 (p = 0.006)	Benefit	Very high
Grant ^58^	Spain	Proxy for radiation (latitude)	Degree of latitude *	Breast cancer	Females	Correlation	r = 0.15 (p > 0.05)	Benefit	n/a
		Behavioural (NMSC mortality and melanoma mortality)	NMSC mortality rate	Breast cancer	Females	Correlation	r = −0.38 (p < 0.01)	Benefit	n/a
			Melanoma mortality rate	Breast cancer	Females	Correlation	r = 0.30 (p < 0.05)	Harm	n/a
Grant ^62^ (1992)	France	Proxy for radiation (latitude)	Degree of latitude *	Breast cancer	Females	Correlation	r = 0.69 (p = 0.001)	Benefit	n/a
Grant ^62^ (1998–2000)	France	Proxy for radiation (latitude)	Degree of latitude squared *	Breast cancer	Females	Correlation	r = 0.66 (p = 0.001)	Benefit	n/a

Note. *An increase in latitude indicates a decrease in sunlight exposure. Therefore, a positive relationship between latitude and mortality suggests a protective effect of sunlight. Abbreviations: b: regression beta coefficient; CI: confidence interval; n/a: not applicable; NMSC: non-melanoma skin cancer; r: correlation coefficient; RoB: risk of bias; UVB: ultraviolet B radiation.

**Figure 7.**
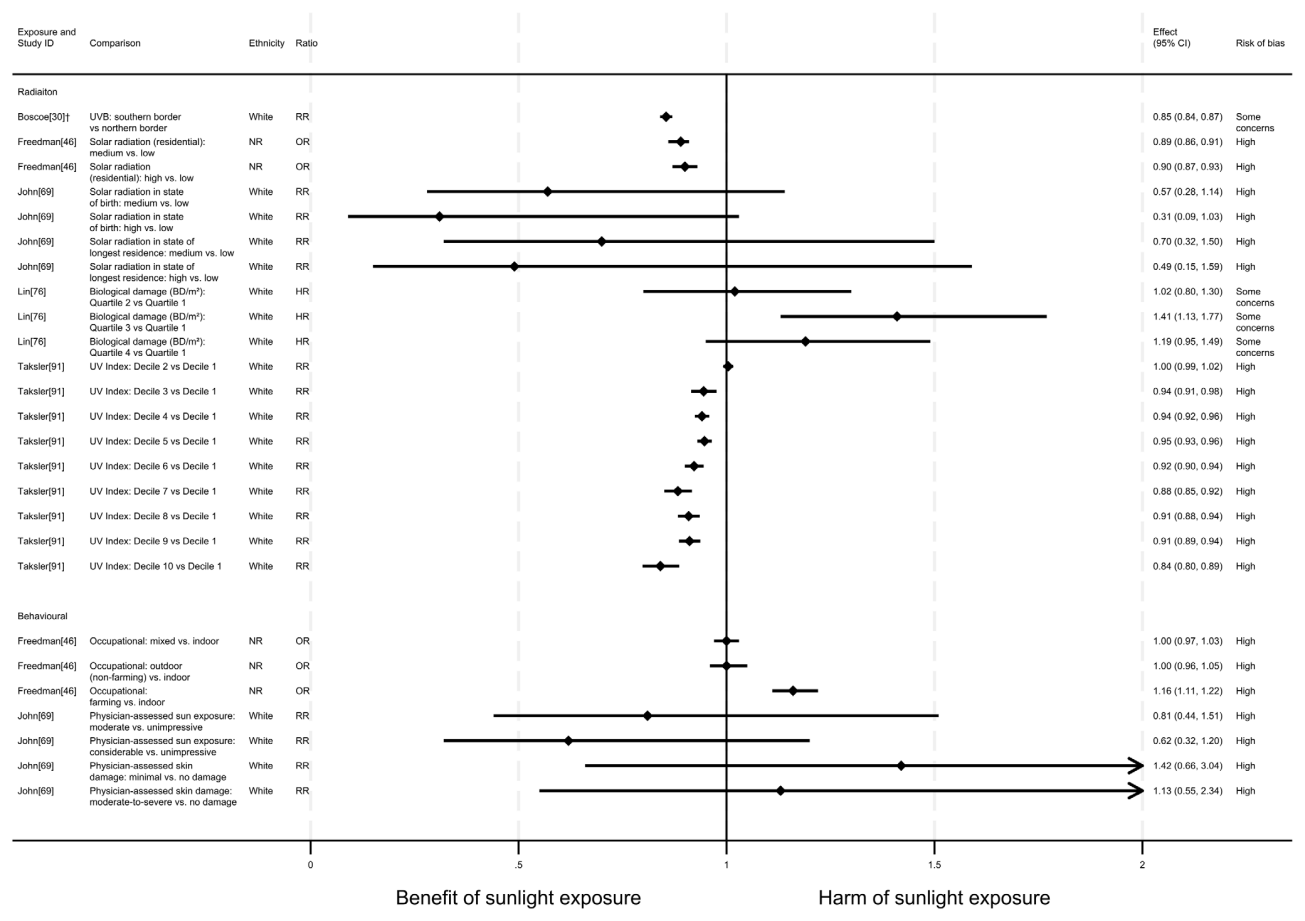
Forest plot of studies showing the effect of exposure on prostate cancer mortality. †Result inverted in order to reflect an increase in exposure. NR: ethnicity of population not reported.

***Prostate cancer mortality.*** There were 15 articles looking at the effect of sunlight on prostate cancer mortality, with some reporting multiple exposure types and/or date ranges (25 analyses reported across the 15 articles). There were 12 articles with an ecological design, two used a cohort and one used a case-control design. The findings were mixed ( Figure 7 and Table 4). Fourteen analysis results looked at the effect of radiation, five looked at proxy radiation measures and six examined the effect of sunlight exposure behaviour. Most results suggested there may be a beneficial effect of sunlight on prostate cancer mortality (14 analyses). However, six results from Lin *et al.* ^76^, Grant and Garland ^56^, Freedman *et al.* ^46^, John *et al.* ^69^ and Grant ^58^, indicated that sunlight may have a harmful effect on prostate cancer mortality. Three results from Grant ^58^, Grant and Garland ^56^ and Mizoue ^80^, found little evidence of an effect; one result from Colli and Grant ^37^ produced very wide confidence intervals compatible with both a benefit and harm and one article ^44^ did not report the direction of effect.

**Table 4.** Linear regression, correlation and narrative results showing the effect of exposure on prostate cancer mortality.

Study	Location	Exposure	Unit of analysis	Outcome	Subgroup	Analysis	Results	Direction of effect	Risk of bias
Colli and Colli ^36^	Worldwide	Radiation (UV index)	UV index unit	Prostate cancer	Males	Regression	β = −0.81 (95% CI −1.292 to −0.322)	Benefit	High
		Proxy for radiation (latitude)	Degree of latitude *	Prostate cancer	Males	Regression	β = 0.14 (95% CI 0.044 to 0.24)	Benefit	High
Colli and Grant ^37^	USA	Radiation (UV index)	UV index unit	Prostate cancer	Black males	Regression	β = 0.2 (95% CI −2.4 to 2.8)	Harm	High
Fleischer and Fleischer ^44^	USA	Radiation (solar radiation)	kJ/m ^2^	Prostate cancer	n/a	Narrative	“No associations were demonstrated between solar energy and cancer mortality for prostate cancer (p = 0.90)”	NR	n/a
Grant ^55^	Worldwide	Radiation (UVB)	MJ/m ^2^ / year	Prostate cancer	Predominantly Caucasian populations	Narrative	Inverse relationship between UV and mortality t = -5.8, p < 0.001	Benefit	n/a
Grant and Garland ^56^ (1950–1969)	USA	Radiation (UVB)	kJ/m ^2^	Prostate cancer	White males	Regression	β = 0.02 (p = 0.94)	Harm	Some concerns
		Proxy for radiation (latitude)	Degree of latitude *	Prostate cancer	White males	Regression	β = 0.52 (p = 0.09)	Benefit	High
Grant and Garland ^56^ (1970–1994)	USA	Radiation (UVB)	kJ/m ^2^	Prostate cancer	White males	Regression	β = 0.38 (p = 0.04)	Harm	Some concerns
		Proxy for radiation (latitude)	Degree of latitude *	Prostate cancer	White males	Regression	β = 0.27 (p = 0.12)	Benefit	High
Grant ^58^	Spain	Proxy for radiation (latitude)	Degree of latitude *	Prostate cancer	Males	Correlation	r = 0.06 (p > 0.05)	Benefit	n/a
		Behavioural (NMSC mortality; melanoma mortality)	NMSC mortality rate	Prostate cancer	Males	Correlation	r = −0.21 (p > 0.05)	Benefit	n/a
			Melanoma mortality rate	Prostate cancer	Males	Correlation	r = 0.52 (p < 0.01)	Harm	n/a
Grant ^62^	France	Proxy for radiation (latitude)	Degree of latitude squared *	Prostate cancer	Males	Correlation	r = 0.68 (p = 0.001)	Benefit	n/a
Grant ^64^	USA (California)	Behavioural (NMSC mortality)	NMSC mortality rate	Prostate cancer	White males	Regression	β = −0.62 (p = 0.005)	Benefit	High
Mizoue ^80^	Japan	Radiation (solar radiation)	KWh/ Hour/day	Prostate cancer	Males	Correlation	r = −0.07 (p > 0.05)	Benefit	n/a
Santos Arrontes *et al.* ^86^	Spain	Radiation (sunlight)	Hours of sun exposure/year	Prostate cancer	Males	Narrative	“Mortality from prostate cancer presented statistically significant differences, being . . . lower in the areas with the greatest number of hours of sunshine per year (p = 0.041).	Benefit	n/a

Note. *An increase in latitude indicates a decrease in sunlight exposure. Therefore, a positive relationship between latitude and mortality suggests a protective effect of sunlight. Abbreviations: b: regression beta coefficient; CI: confidence interval; n/a: not applicable; NMSC: non-melanoma skin cancer; NR: not reported r: correlation coefficient; RoB: risk of bias; UV: ultraviolet radiation; UVB: ultraviolet B radiation.

**Figure 8.**
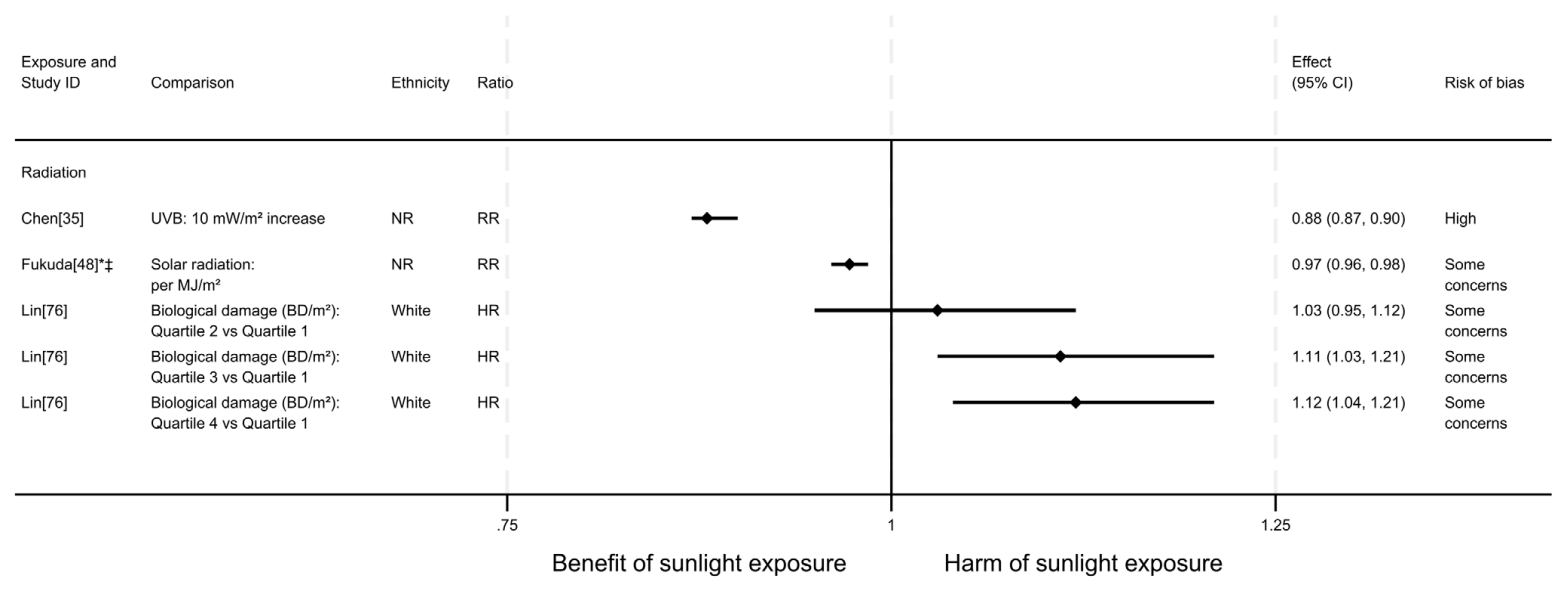
Forest plot of studies showing the effect of exposure on lung cancer mortality. *Fixed-effects meta-analysis performed to combine gender subgroups. ‡Ratio calculated via exponentiating logistic regression beta coefficient. NR: ethnicity of population not reported.

***Lung cancer mortality.*** There were 10 articles investigating the effect of sunlight on lung cancer mortality, with some reporting multiple exposure types (12 analyses reported across the 10 articles). Nine articles had an ecological design and one had a cohort design. There were six analyses looking at the effect of radiation, three looked at proxy radiation measures and three looked at sunlight exposure behaviour. The findings were mixed ( Figure 8 and Table 5). The majority of analyses (n = 7), reported in Chen *et al.* ^35^, Fukuda *et al.* ^48^, Fleischer and Fleischer ^44^, Grant ^57, 59, 64^, and Garland *et al.* ^50^ found that higher levels of sunlight were associated with a decreased risk of lung cancer mortality. However, three analyses in Lin *et al.* ^76^ and Grant ^58^ suggested that there may be a harmful effect of sunlight. The findings of two analyses from two articles ^58, 62^ were mixed, with different findings reported for males and females.

**Table 5.** Linear regression, correlation and narrative results showing the effect of exposure on lung cancer mortality.

Study	Location	Exposure	Unit of analysis	Outcome	Subgroup	Analysis	Results	Direction of effect	RoB
Fleischer and Fleischer ^44^	USA	Radiation (solar radiation)	kJ/m ^2^	Lung cancer	n/a	Narrative	“Associations were demonstrated between increasing solar energy and decreasing cancer incidence for lung cancer (p < 0.001)”	Benefit	n/a
Garland *et al.* ^50^	USA	Radiation (solar radiation)	Calories/cm²	Lung, trachea and bronchus cancer	Females	Correlation	r = −0.19 (p = 0.28)	Benefit	n/a
Grant ^57^	USA	Radiation (UVB DNA)	kJ/m ^2^	Lung cancer	Black males	Regression	β = −0.52 (p = 0.003)	Benefit	Very high
					Black females	Regression	β = −0.29 (p = 0.08)	Benefit	Very high
Grant ^58^	Spain	Proxy for radiation (latitude)	Degree of latitude *	Lung cancer	Males	Correlation	r = −0.36 (p < 0.05)	Harm	n/a
					Females	Correlation	r = −0.05 (p > 0.05)	Harm	n/a
		Behavioural (NMSC mortality; melanoma mortality)	NMSC mortality rate	Lung cancer	Males	Correlation	r = 0.02 (p > 0.05)	Mixed (gender)	n/a
					Females	Correlation	r = −0.31 (p < 0.05)	Mixed (gender)	n/a
			Melanoma mortality rate	Lung cancer	Males	Correlation	r = 0.33 (p < 0.05)	Harm	n/a
					Females	Correlation	r = 0.36 (p < 0.05)	Harm	n/a
Grant ^59^	China	Proxy for radiation (latitude)	Degree of latitude *	Lung cancer	35–64 year old males	Regression	β = 0.43 (p = 0.002)	Benefit	High
Grant ^62^	France	Proxy for radiation (latitude)	Degree of latitude squared *	Lung cancer	Males	Correlation	r = 0.54 (p = 0.01)	Benefit	n/a
					Females	Narrative	Not significant	Mixed (gender)	n/a
Grant ^64^	USA (California)	Behavioural (NMSC mortality)	NMSC mortality rate	Lung cancer	White males	Regression	β = −0.38 (p = 0.11)	Mixed (gender)	High

Note. *An increase in latitude indicates a decrease in sunlight exposure. Therefore, a positive relationship between latitude and mortality suggests a protective effect of sunlight. Abbreviations: b: regression beta coefficient; n/a: not applicable; NMSC: non-melanoma skin cancer; NR: not reported r: correlation coefficient; RoB: risk of bias; UVB DNA: DNA-weighted UVB radiation.

**Figure 9.**
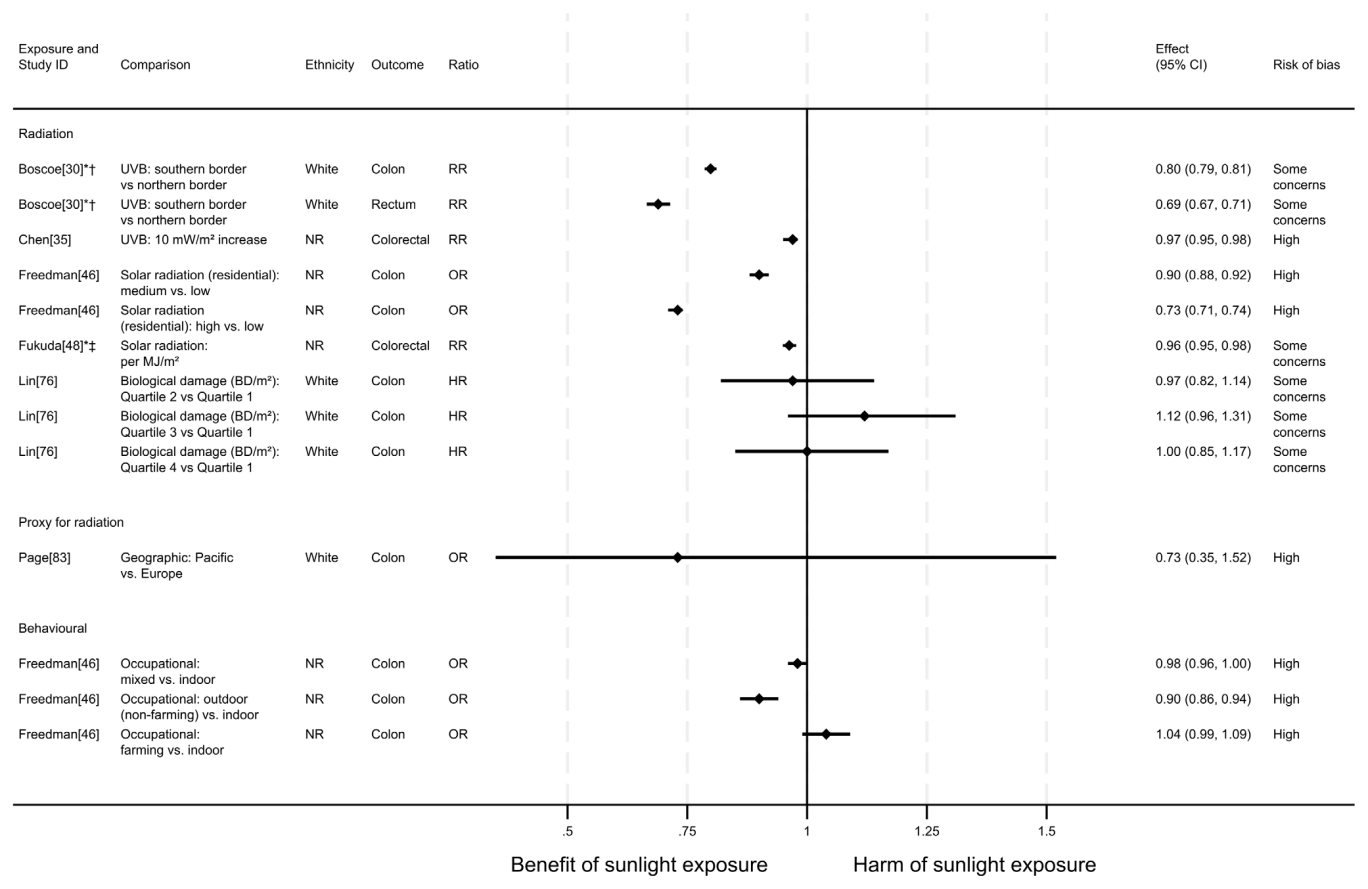
Forest plot of studies showing the effect of exposure on bowel cancer mortality. *Fixed-effects meta-analysis performed to combine gender subgroups. †Result inverted in order to reflect an increase in exposure. ‡Ratio calculated via exponentiating logistic regression beta coefficient. NR: ethnicity of population not reported.

***Bowel cancer mortality.*** There were 14 articles measuring the effect of sunlight on bowel cancer mortality, with some reporting multiple exposure types, outcomes and/or date ranges (31 analyses reported across the 14 articles). There were 11 articles with an ecological design, two used a cohort and one had case-control design. Sixteen analysis results examined radiation measures, eight looked at proxy radiation measures and seven looked at the effect of exposure behaviour measures. Overall, the majority of analysis results (n = 22) suggested that higher levels of sunlight were associated with a decreased risk of bowel cancer mortality ( Figure 9 and Table 6). However, four analyses from Grant ^58^ and Veach *et al.* ^94^ indicated that there may be a harmful association between sunlight and mortality. Three analyses from Grant ^58^, Lin *et al.* ^76^ and Freedman *et al.* ^46^ produced mixed findings, with conflicting results found either across dose levels or between gender. The result in Page *et al.* ^83^ produced very wide confidence intervals that were compatible with both a benefit and a harm, whilst in Fleischer and Fleischer ^44^ the direction of effect was not reported.

**Table 6.** Linear regression, correlation and narrative results showing the effect of exposure on bowel cancer mortality.

Study	Location	Exposure	Unit of analysis	Outcome	Subgroup	Analysis	Results	Direction of effect	RoB
Fleischer and Fleischer ^44^	USA	Radiation (solar radiation)	kJ/m ^2^	Colon/rectum cancer	n/a	Narrative	“No associations were demonstrated between solar energy and cancer mortality for colon/ rectum cancer (p = 0.12)”	NR	n/a
Grant and Garland ^56^ (1950–1969)	USA	Radiation (UVB)	kJ/m ^2^	Colon cancer	White males	Regression	β = −0.63 (p < 0.001)	Benefit	Some concerns
					White females	Regression	β = −0.70 (p < 0.001)	Benefit	Some concerns
				Rectum cancer	White males	Regression	β = −0.62 (p < 0.001)	Benefit	Some concerns
					White females	Regression	β = −0.65 (p < 0.001)	Benefit	Some concerns
Grant and Garland ^56^ (1970–1994)	USA	Radiation (UVB)	kJ/m ^2^	Colon cancer	White males	Regression	β = −0.71 (p < 0.001)	Benefit	Some concerns
					White females	Regression	β = −0.76 (p < 0.001)	Benefit	Some concerns
				Rectum cancer	White males	Regression	β = −0.75 (p < 0.001)	Benefit	Some concerns
					White females	Regression	β = −0.70 (p < 0.001)	Benefit	Some concerns
Grant ^57^	USA	Radiation (UVB DNA)	kJ/m ^2^	Colon cancer	Black males	Regression	β = −0.37 (p = 0.03)	Benefit	Very high
					Black females	Regression	β = −0.05 (p = 0.76)	Benefit	Very high
				Rectum cancer	Black males	Regression	β = −0.38 (p = 0.02)	Benefit	Very high
					Black females	Regression	β = −0.02 (p = 0.94)	Benefit	Very high
Grant ^58^	Spain	Proxy for radiation (latitude)	Degree of latitude *	Colon cancer	Males	Correlation	r = 0.19 (p > 0.05)	Mixed (gender)	n/a
					Females	Correlation	r = −0.006 (p > 0.05)	Mixed (gender)	n/a
				Rectum cancer	Males	Correlation	r = 0.61 (p < 0.01)	Benefit	n/a
					Females	Correlation	r = 0.33 (p < 0.05)	Benefit	n/a
		Behavioural (NMSC mortality; melanoma mortality)	NMSC mortality rate	Colon cancer	Males	Correlation	r = −0.30 (p < 0.05)	Benefit	n/a
					Females	Correlation	r = −0.31 (p < 0.05)	Benefit	n/a
				Rectum cancer	Males	Correlation	r = −0.46 (p < 0.01)	Benefit	n/a
					Females	Correlation	r = −0.35 (p < 0.05)	Benefit	n/a
			Melanoma mortality rate	Colon cancer	Males	Correlation	r = 0.47 (p < 0.01)	Harm	n/a
					Females	Correlation	r = 0.43 (p < 0.01)	Harm	n/a
				Rectum cancer	Males	Correlation	r = 0.31 (p < 0.05)	Harm	n/a
					Females	Correlation	r = 0.26 (p > 0.05)	Harm	n/a
Grant ^59^	China	Proxy for radiation (latitude)	Degree of latitude *	Colorectal cancer	35–64 years old males	Regression	β = 0.41 (p = 0.005)	Benefit	High
Grant ^62^ (1992)	France	Proxy for radiation (latitude)	Degree of latitude *	Colorectal cancer	Males	Correlation	r = 0.53 (p = 0.01)	Benefit	n/a
					Females	Correlation	r = 0.46 (p = 0.04)	Benefit	n/a
Grant ^62^ (1998–2000)	France	Proxy for radiation (latitude)	Degree of latitude squared *	Colorectal cancer	Males	Correlation	r = 0.49 (p = 0.02)	Benefit	n/a
					Females	Correlation	r = 0.65 (p = 0.001)	Benefit	n/a
Grant ^64^	USA (California)	Proxy for radiation (latitude)	Degree of latitude *	Colon cancer	White males	Regression	β = 0.47 (p = 0.01)	Benefit	High
				Rectum cancer	White males	Regression	β = 0.48 (p = 0.007)	Benefit	High
		Behavioural (NMSC mortality)	NMSC mortality rate	Colon cancer	White males	Regression	β = −0.64 (p = 0.002)	Benefit	High
				Rectum cancer	White males	Regression	β = −0.48 (p = 0.009)	Benefit	High
Veach *et al.* ^94^	USA	Radiation (solar radiation)	Weber/m ^2^	Colorectal cancer	Black males	Regression	β = 0.003 (p < 0.001)	Harm	Some concerns
					White males	Regression	β = −0.001 (p = 0.001)	Benefit	Some concerns
					Hispanic males	Regression	β = 0.001 (p = 0.033)	Harm	Some concerns

Note. *An increase in latitude indicates a decrease in sunlight exposure. Therefore, a positive relationship between latitude and mortality suggests a protective effect of sunlight. Abbreviations: b: regression beta coefficient; n/a: not applicable; NMSC: non-melanoma skin cancer; NR: not reported r: correlation coefficient; RoB: risk of bias; UVB: ultraviolet B radiation; UVB DNA: DNA-weighted UVB radiation.

**Figure 10.**
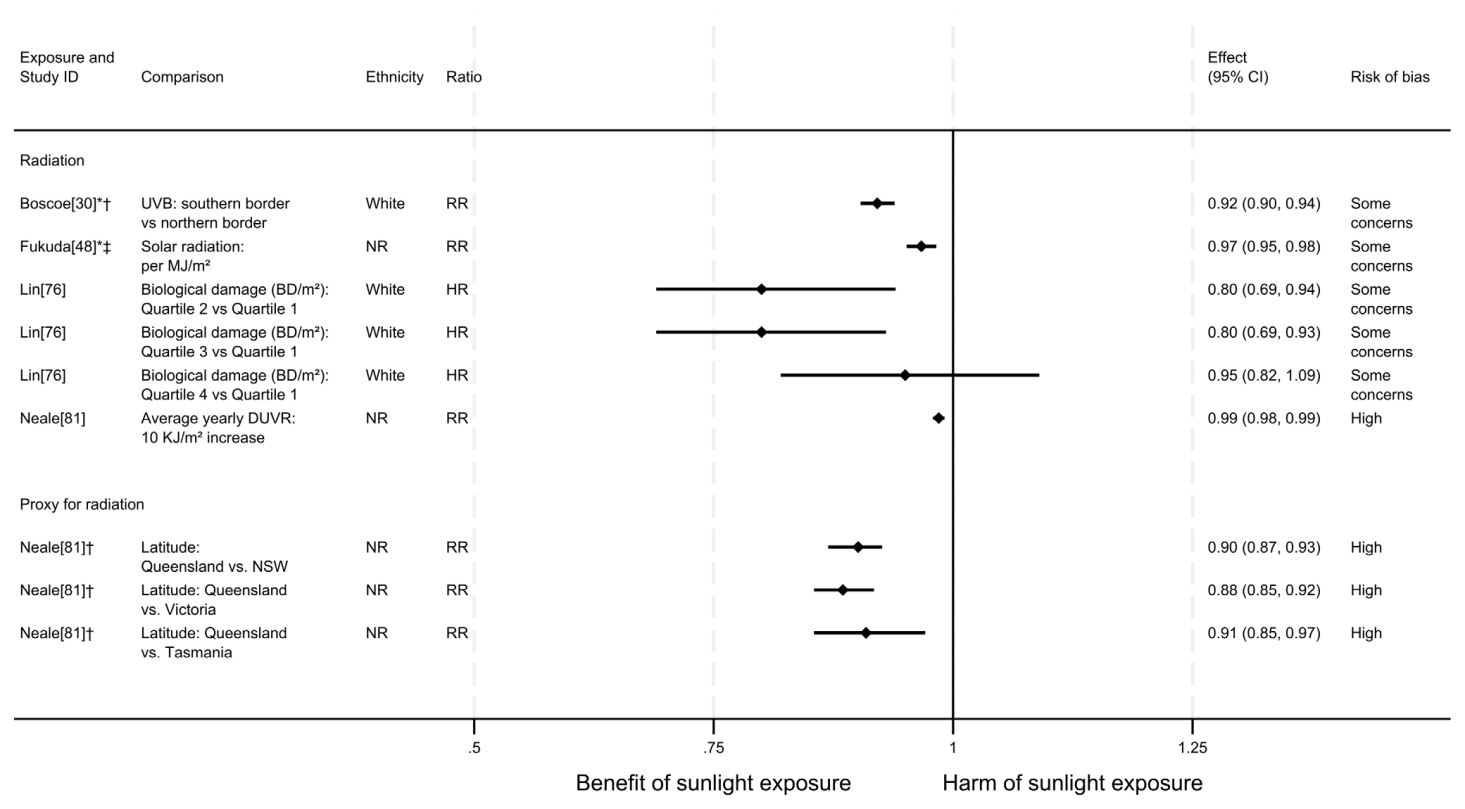
Forest plot of studies showing the effect of exposure on pancreatic cancer mortality. *Fixed-effects meta-analysis performed to combine gender subgroups. †Result inverted in order to reflect an increase in exposure. ‡Ratio calculated via exponentiating logistic regression beta coefficient. NR: ethnicity of population not reported.

***Pancreatic cancer mortality.*** There were seven articles measuring the effect of sunlight on pancreatic cancer mortality, with some reporting multiple exposure types or date ranges (11 analyses reported across the seven articles). Six articles had an ecological design and one used a cohort. There were seven analyses examining the effect of radiation, two articles looked at proxy for radiation measures and two looked at sunlight exposure behaviours. The majority of analyses (n = 9) suggested that higher levels of sunlight are associated with a decreased risk of pancreatic cancer mortality, as reported in Boscoe and Schymura ^30^, Fukuda *et al.* ^48^, Lin *et al.* ^76^, Neale *et al.* ^81^, Grant and Garland ^56^, and Grant ^58^. However, one analysis in Grant ^58^ indicated there may be a harmful association between melanoma mortality rates and pancreatic cancer mortality. One analysis in Fleischer and Fleischer ^44^ was reported narratively, without a direction of effect ( Figure 10 and Table 7).

**Table 7.** Linear regression, correlation and narrative results showing the effect of exposure on pancreatic cancer mortality.

Study	Location	Exposure	Unit of analysis	Outcome	Subgroup	Analysis	Results	Direction of effect	RoB
Fleischer and Fleischer ^44^	USA	Radiation (solar radiation)	kJ/m ^2^	Pancreas cancer	n/a	Narrative	“No associations were demonstrated between solar energy and cancer mortality for pancreatic cancer (p = 0.07)”	NR	n/a
Grant and Garland ^56^ (1950–1969)	USA	Radiation (UVB)	kJ/m ^2^	Pancreas cancer	White males	Regression	β = −0.39 (p = 0.02)	Benefit	Some concerns
					White females	Regression	β = −0.74 (p < 0.001)	Benefit	Some concerns
Grant and Garland ^56^ (1970–1994)	USA	Radiation (UVB)	kJ/m ^2^	Pancreas cancer	White males	Regression	β = −0.46 (p = 0.005)	Benefit	Some concerns
					White females	Regression	β = −0.34 (p = 0.06)	Benefit	Some concerns
Grant ^58^	Spain	Proxy for radiation (latitude)	Degree of latitude *	Pancreas cancer	Males	Correlation	r = 0.55 (p < 0.01)	Benefit	n/a
					Females	Correlation	r = 0.40 (p < 0.01)	Benefit	n/a
		Behavioural (NMSC mortality; melanoma mortality)	NMSC mortality rate	Pancreas cancer	Males	Correlation	r = −0.35 (p < 0.05)	Benefit	n/a
					Females	Correlation	r = −0.35 (p < 0.05)	Benefit	n/a
			Melanoma mortality rate	Pancreas cancer	Males	Correlation	r = 0.24 (p > 0.05)	Harm	n/a
					Females	Correlation	r = 0.45 (p < 0.01)	Harm	n/a

Note. *An increase in latitude indicates a decrease in sunlight exposure. Therefore, a positive relationship between latitude and mortality suggests a protective effect of sunlight. Abbreviations: b: regression beta coefficient; n/a: not applicable; NMSC: non-melanoma skin cancer; NR: not reported r: correlation coefficient; RoB: risk of bias; UVB: ultraviolet B radiation.

**Figure 11.**
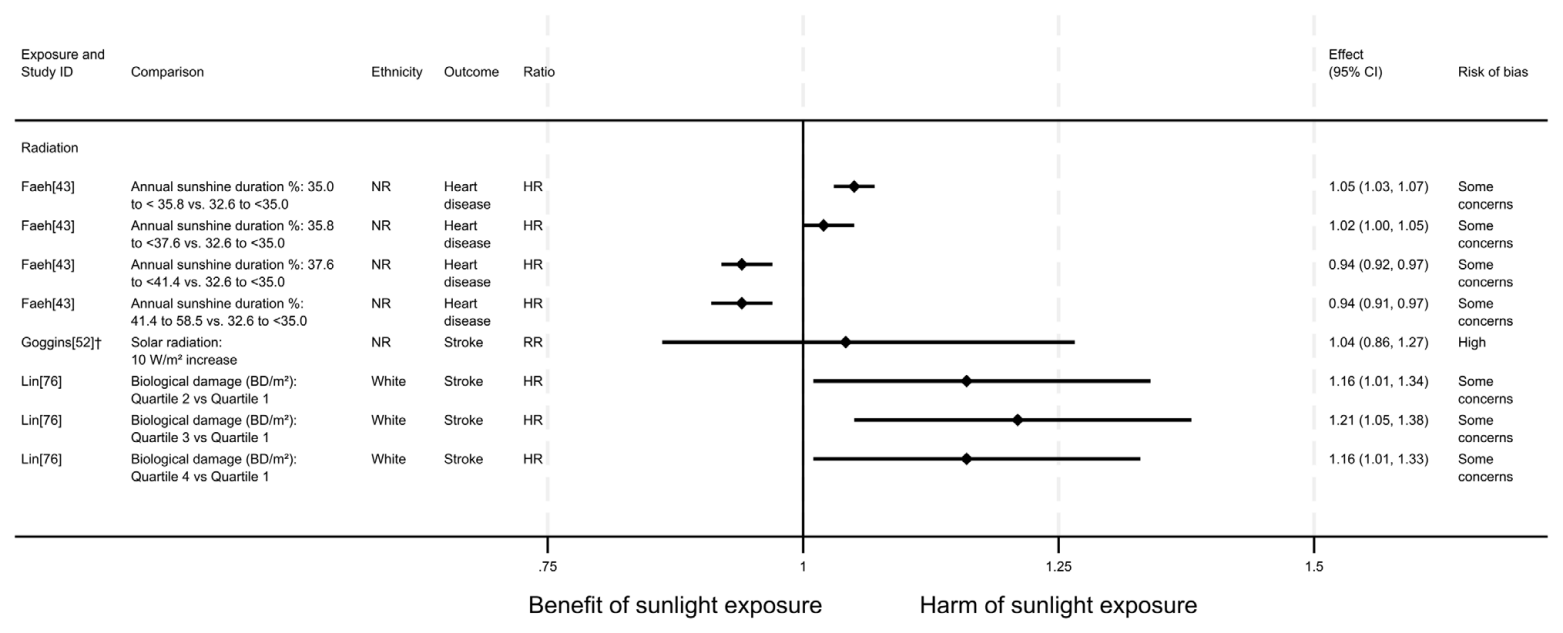
Forest plot of studies showing the effect of exposure on cause-specific CVD mortality. †Result inverted in order to reflect an increase in exposure. NR: ethnicity of population not reported.

***Cause-specific CVD mortality.*** Six articles looked at cause-specific CVD mortality (7 analyses across the 6 articles); four with an ecological design and two using a cohort design. All seven analyses examined radiation measures. Four results looked at the effect on heart disease mortality and three looked at the effect on stroke mortality. The findings were mixed ( Figure 11 and Table 8). Two analyses suggested a beneficial effect of radiation on heart disease mortality ^42, 87^. However, three analyses reported a harmful effect on stroke mortality ^42, 52, 76^. One produced mixed results ^43^, and one did not report a direction of effect ^34^ ( Figure 11 and Table 8).

**Table 8.** Linear regression, correlation and narrative results showing the effect of exposure on cause-specific CVD mortality.

Study	Location	Exposure	Unit of analysis	Outcome	Subgroup	Analysis	Results	Direction of effect	RoB
Camara and Brandao ^34^	Worldwide	Radiation (Solar incidence)	kWh/m ^−2^ / day ^−1^	Coronary heart disease	n/a	Narrative	No significant difference between high and low sunlight incidence countries (p > 0.05)	NR	n/a
Ezzati *et al.* ^42^	USA	Radiation (insolation)	NR	Ischaemic heart disease	45+ year old males	Regression	β = −0.00023 (95% CI −0.0011 to 0.00061)	Benefit	Some concerns
					45+ year old females	Regression	β = −0.00014 (95% CI −0.00099 to 0.00072)	Benefit	Some concerns
				Stroke	45+ year old males	Regression	β = 0.00046 (95% CI 0.0002 to 0.00072)	Harm	Some concerns
					45+ year old females	Regression	β = 0.00062 (95% CI 0.00025 to 0.00099)	Harm	Some concerns
Scarborough *et al.* ^87^	UK	Radiation (sunshine)	1000s hours/ year	Coronary heart disease	Males	Regression	β = −27.3 (p < 0.05)	Benefit	Some concerns
					Females	Regression	β = −14.3 (p < 0.05)	Benefit	Some concerns

Abbreviations: b: regression beta coefficient; CI: confidence interval; n/a: not applicable; NR: not reported r: correlation coefficient; RoB: risk of bias.

### Subgrouping by skin type/colour or ethnicity

We sought to investigate differences in effect between people with different skin types/colours or ethnicity. However, information to allow this was limited. Most articles reported findings that examined the whole population (62%), and several limited their population to White people only (24%).

One study in the USA examined all-CVD mortality by ethnicity subgroup ^26^, reporting slightly higher mortality risk associated with sunlight exposure for White, Black, Hispanic and Asian people, but a slightly lower risk among Native Americans. Also in the USA, Boscoe and Schymura ^30^ found that residing along the southern border (erythemally-weighted UVB exposure of roughly 1540 kJ/m ^2^/year) was associated with a decreased risk of breast cancer mortality compared with residing along the northern border (roughly 650 kJ/m ^2^/year) among both White women (RR = 0.87, 95% CI 0.85 to 0.88) and Black women (RR = 0.90, 95% CI 0.86 to 0.94). Pennello *et al.* ^84^ found a harmful relationship between UVB and both melanoma and NMSC mortality for both Black and White people. Finally, Veach *et al.* ^94^ found that higher solar radiation (Weber/m ^2^ by state) was associated with higher risk of bowel cancer mortality in Black (β = 0.003, p < 0.001) and Hispanic men (β = 0.001, p = 0.033), but lower risk in White men (β = −0.001, p = 0.001).

It was sometimes possible to make indirect comparisons across people of different skin types/colours or ethnicity. Grant and Garland ^56^ and Grant ^57^ reported data for the White population and the Black population, respectively, in the USA between 1970 and 1994. The results were suggestive of a beneficial effect of sunlight on all-cancer, breast cancer and bowel cancer mortality for both Black and White people. Boscoe and Schymura ^30^ reported that higher levels of sunlight exposure were associated with a decreased risk of prostate cancer in White men in the USA between 1993 and 2002 (RR = 0.85, 95% CI 0.84 to 0.87); whilst conversely, Colli and Grant ^37^ observed a small positive association between higher winter UV Index and prostate cancer mortality among Black men in the USA between 1992 and 2001, though the confidence intervals were compatible with both benefit and harm (β = 0.20; 95% CI −2.4 to 2.8).

## Discussion

The evidence identified by this review provides a mixed message about the association between sunlight exposure and mortality risk. Eight articles reported data for our primary outcome, with half having results in the direction of a beneficial association and half with results in the direction of a harmful association between sunlight and all-cause mortality. Mixed results were also found for all-CVD mortality, while a majority reported that higher levels of sunlight were associated with lower risk of all-cancer mortality. There was considerable uncertainty in the results across all outcomes.

As expected, most articles looking at skin cancer found that higher levels of sunlight were associated with higher levels of both melanoma and NMSC mortality. In contrast, most articles examining the five cancers with the highest UK mortality rate (breast, prostate, lung, bowel and pancreatic cancer) found that higher levels of sunlight were associated with lower risks of mortality. However, for each of these specific cancers the evidence was not fully consistent, with some findings also suggesting a harmful effect of sunlight. There were also mixed findings when looking at specific causes of CVD mortality (heart disease and stroke). As with the primary outcomes, there was considerable uncertainty in the findings.

Many of the associations we observed between higher sunlight exposure and lower risk of all-cause and all-CVD mortality came from studies conducted in higher latitude countries (specifically, the UK and Sweden). On the other hand, many of the studies finding associations between higher sunlight exposure and higher risk of these mortality outcomes were conducted in the USA. These observations are consistent with the possibility that, whilst there are long established risks associated with sunlight exposure in high UVR locations, the benefits of sunlight may possibly outweigh the harms in regions with a generally low UV index ^10^. However, this is not a conclusion we can reach with confidence from the data, given the limitations of the evidence base. Furthermore, a potentially beneficial effect of sunlight on all-cause and all-CVD mortality was observed in Hong Kong ^52^, the location closest to the equator amongst all those included in the analyses.

We intended to examine the extent to which the effects of sunlight exposure on mortality would vary according to skin type/colour or ethnicity. Evidence to allow this investigation was limited. In the four studies that reported results by these subgroups, the findings suggested some differences. For example, in the USA, the Native American population were found to have a more beneficial association between sunlight exposure and all-CVD mortality, compared with other ethnicities ^26^. Though there were also findings suggesting similar results between those of different skin type/colour or ethnicity, such as the beneficial association between UVB and breast cancer mortality found for both Black and White women ^30^.

Around a quarter of the main articles included in this review were restricted to White populations. Around two thirds reported on the whole population, though given that a large number of these were conducted in Europe and North America, it is likely that the populations in those articles were predominantly White as well. In order to gain a more complete picture of the relationship between sunlight exposure and mortality, further studies investigating the impact of skin type/colour or ethnicity are warranted. This, in turn, would allow organizations responsible for sun safety messaging to provide more nuanced guidance for those with different skin types.

While it is well-established that sunlight increases the risk of skin cancer, particularly through UVR damage to skin cell DNA, the mechanisms through which sunlight may affect non-skin cancer risk and mortality are unclear. Sunlight exposure of the skin is usually the body’s major source of vitamin D ^11^ and experimental studies show that vitamin D can potentially slow or prevent the development of cancer by several cellular mechanisms. These include promoting cellular differentiation and cell death, reducing cancer cell division and tumour blood vessel formation, inhibiting tumour progression and metastasis ^98^, and stimulating the immune response to cancer cells ^99^. Moreover, vitamin D receptors are present in many organs and cell types ^100^. It is therefore conceivable that some of the beneficial effects of sunlight on mortality may be mediated by vitamin D. However, in randomized trials, vitamin D supplements had little to no effect on the risk of developing cancers (both overall and at specific sites) as well as on CVD and all-cause mortality ^101^. It is possible, therefore, that the suggested beneficial effects of sunlight on cancer mortality may involve vitamin D-independent pathways.

The radiation measured by studies in this review included solar radiation (encompassing UV, visible and infrared radiation) and ambient UVR (UVB and UVA), while many focused primarily on UVB, which encompasses the wavelengths initiating vitamin D synthesis in the skin, as well as being principally responsible for direct DNA damage in skin cells. However, it is now recognized that there is a range of potential benefits of solar radiation on health besides vitamin D synthesis ^102^. The radiation responsible for these effects may include UVB and/or UVA, and possibly other types of radiation. For example, UVA and UVB are reported to regulate release of the vasodilator nitric oxide from skin cells, potentially protecting against CVD ^15, 16^. While UVB is generally more potent than UVA in effecting local skin immunomodulation, both may influence systemic immunity ^100^. Therefore, it is warranted to consider a broader range of solar radiation and its effects with respect to mortality, than UVB alone.

Strengths of our review include our pre-specified review methodology with a comprehensive search and adoption of systematic review methods widely considered to reduce biases and human error. We included a wide range of measures of sunlight exposure, including measurements of radiation, proxy measures of radiation, and behaviour associated with sunlight exposure. Since we might expect the nature of confounding to be different for different exposures (e.g. for geographical level versus individual level exposure measures), our approach builds in the idea of ‘triangulating’ analyses that have different potential biases ^103^. Our comprehensive approach enabled enquiry into an increasingly debated area of public health, i.e. the benefits and risks of sunlight exposure, through assessment of associations with all-cause mortality, and separately with all-cancer mortality and all-CVD mortality. We found a large number of articles and have presented the results systematically by outcome, with consideration of risk of bias, consistency of findings and imprecision.

There was considerable potential for bias in the results of the included studies. None of the results included in this review were judged to be at low risk of bias, and, in most cases, we judged the results to be at high risk of bias. The main sources of bias were a lack of control over important confounders, and the measurement of sunlight exposure. There is also a high risk of publication bias, with the potential for statistically significant associations (potentially in either direction) being considered more attractive for publication.

Concerns about indirectness (or applicability) of the evidence arose primarily from the measures of exposure. The measures of radiation encompassed heterogenous methodology and measurement units, ranging from satellite-derived data and ground-based measurements of radiation, to mean annual sunshine hours. Some of these measures can provide reliable estimates of ambient sunlight levels at a given location, but do not provide information about individual-level or skin exposure, which are influenced by sun exposure behaviour and sun protection measures (e.g. use of clothing, sunscreens and shade).

Some of the findings were imprecise, with wide margins of error which were often compatible with a higher and lower risk of mortality being associated with higher sunlight exposure. There was also inconsistency in the findings. For every outcome included in the review, we found results suggesting both a beneficial and a harmful effect of sunlight.

In conclusion, evidence from existing observational epidemiological studies of the association between sunlight exposure and mortality is inconclusive. While most studies of skin cancer mortality demonstrate a higher risk associated with more exposure to sunlight, many studies of other cancers have reported lower risks associated with more exposure to sunlight. Evidence for cardiovascular mortality is mixed. Perhaps because of a variety of effects that sunlight can have, or possibly because of potential biases in the studies available, findings for overall mortality are too variable to provide a rationale for changes to sun protection guidance.

## Data Availability

Figshare: Dataset for “The effects of sunlight exposure on mortality: a systematic review of epidemiological studies” https://doi.org/10.6084/m9.figshare.28660109 ^104^. The project contains the following underlying data: Dataset - effects of sunlight on mortality.xlsx Data are available under the terms of the Creative Commons Attribution 4.0 International license (CC-BY 4.0). Figshare: Supplementary information for ““The effects of sunlight exposure on mortality: a systematic review of epidemiological studies” https://doi.org/10.6084/m9.figshare.29069657 ^21^ This project contains the following extended data: Appendix S1. Search strategies. Table S1. Characteristics of included studies. Table S2. Overlapping data and main article selection. Table S3. Description of exposures measured in articles included in analysis. Table S4. Risk-of-bias assessments. Data are available under the terms of the Creative Commons Attribution 4.0 International license (CC-BY 4.0). Figshare: PRISMA checklist for "The effects of sunlight exposure on mortality: a systematic review of epidemiological studies" https://doi.org/10.6084/m9.figshare.28937123 ^105^. Data are available under the terms of the Creative Commons Attribution 4.0 International license (CC-BY 4.0).
