# Supplementary material for: The effects of sunlight exposure on mortality: a systematic review of epidemiological studies
Source: NIHR Open Res. 2025 Nov 28;5:51. Originally published 2025 Jun 18. [Version 2] doi: 10.3310/nihropenres.13980.2 (PMC12709114; doi:10.3310/nihropenres.13980.2)
Supplement: Peer review history

# Reviewer report: Reviewer response for version 2

**DOI:** 10.3310/nihropenres.15427.r38570

**Published:** 2026-01-08

**Authors:** Douglas Brash

**Affiliations:** 1 Yale School of Medicine, New Haven, USA

Despite the statement in Summary of Revisions that "The figures have all been amended in order that the x-axis now represents the exponential scale", I see no changes from v.1. It is still the case that a 2-fold decrease in harm (0.5x) is given the same space on the axis as a 1.5-fold increase in harm, visually underestimating the magnitude of the beneficial effect. My Approval Status remains the same.

Are the rationale for, and objectives of, the Systematic Review clearly stated?

Yes

Is the statistical analysis and its interpretation appropriate?

I cannot comment. A qualified statistician is required.

Are sufficient details of the methods and analysis provided to allow replication by others?

Yes

Are the conclusions drawn adequately supported by the results presented in the review?

Yes

Reviewer Expertise:

Biophysics, molecular genetics, and cell biology of sunlight-induced skin cancer; photobiology.

I confirm that I have read this submission and believe that I have an appropriate level of expertise to confirm that it is of an acceptable scientific standard, however I have significant reservations, as outlined above.

# Reviewer report: Reviewer response for version 2

**DOI:** 10.3310/nihropenres.15427.r38571

**Published:** 2026-01-06

**Authors:** Leonardo Vinícius Monteiro de Assis

**Affiliations:** 1 University of Lübeck, Lübeck, Germany; 2 Chemistry and Molecular Biology, University of Gothenburg, Gothenburg, Västra Götaland County, Sweden

The authors have addressed all my concerns in the revised version. I have no further comments.

Are the rationale for, and objectives of, the Systematic Review clearly stated?

Yes

Is the statistical analysis and its interpretation appropriate?

Yes

Are sufficient details of the methods and analysis provided to allow replication by others?

Yes

Are the conclusions drawn adequately supported by the results presented in the review?

Yes

Reviewer Expertise:

I'm experienced in the fields of skin biology, circadian biology, photobiology, and metabolism.

I confirm that I have read this submission and believe that I have an appropriate level of expertise to confirm that it is of an acceptable scientific standard.

# Reviewer report: Reviewer response for version 2

**DOI:** 10.3310/nihropenres.15427.r38574

**Published:** 2025-12-30

**Authors:** Richard Weller

**Affiliations:** 1 University of Edinburgh, Edinburgh, UK

thanks for incorporating our comments on the overall structure and analysis into the revised text which have improved the review. 

 My one final comment relates specifically to the Edinburgh paper 'Stevenson et al (89)' which is classified (Fig 2, 3 and 4) as only using latitude as a proxy for radiation.   This  misses out the important fact that it also used a second independent and very different measure,  sunbed use, as  a behavioural measure for sunlight/UV exposure.    Concordant inverse associations of both exposures with all cause, cardiovascular, and non-skin cancer mortality—alongside the expected positive association with melanoma and non-melanoma skin cancer incidence—form a triangulation that is difficult to ascribe to a single omitted confounder. More broadly, the authors explicitly employed triangulation—using two operationally distinct exposures with different confounding structures—to test whether concordant findings persist across independent sources of bias, thereby strengthening causal interpretation.  They prespecified and observed null associations with a negative control outcome (hospitalisations due to car/motorcycle accidents), arguing against unmeasured risk taking or socioeconomic bias driving our results. They also validated exposure against serum 25(OH)D, with appropriate caveats.

 Given this robust epidemiological approach, I am surprised that this paper was rated as being at high risk of bias. As the paper is one of the largest in your review, this may well affect your weighting of evidence.

Are the rationale for, and objectives of, the Systematic Review clearly stated?

Yes

Is the statistical analysis and its interpretation appropriate?

Partly

Are sufficient details of the methods and analysis provided to allow replication by others?

Yes

Are the conclusions drawn adequately supported by the results presented in the review?

No

Reviewer Expertise:

Dermatology, photobiology, epidemiology, translational medicine

I confirm that I have read this submission and believe that I have an appropriate level of expertise to confirm that it is of an acceptable scientific standard, however I have significant reservations, as outlined above.

# Reviewer report: Reviewer response for version 2

**DOI:** 10.3310/nihropenres.15427.r38575

**Published:** 2025-12-30

**Authors:** Rasha Shraim

**Affiliations:** 1 Trinity College Dublin, Dublin, Ireland

I have reviewed the authors' changes and have no further comments.

Are the rationale for, and objectives of, the Systematic Review clearly stated?

Yes

Is the statistical analysis and its interpretation appropriate?

Yes

Are sufficient details of the methods and analysis provided to allow replication by others?

Yes

Are the conclusions drawn adequately supported by the results presented in the review?

Partly

Reviewer Expertise:

Epidemiology, genetic epidemiology, sun exposure and vitamin D

I confirm that I have read this submission and believe that I have an appropriate level of expertise to confirm that it is of an acceptable scientific standard.

# Reviewer report: Reviewer response for version 2

**DOI:** 10.3310/nihropenres.15427.r38572

**Published:** 2025-12-16

**Authors:** Richard McKenzie, Ben Liley, James Liley

**Affiliations:** 1 National Institute of Water and Atmospheric Research Lauder Atmospheric Research Station (Ringgold ID: 563277), Lauder, Otago, New Zealand; 2 Mathematical Sciences, University of Durham, Durham, UK

We’re now happy for the paper to be accepted.

 We would however suggest a change to the (rather surprising) text added on page 18, to emphasise that it’s the UV, rather than the total sunlight that will be different. There’ll less exposure too because of the additional clothing there.  I also wonder if lower temperatures, or lighter skin colourings (perhaps associated with summer holidays at lower latitudes), may be factors. Those points could also be added.

 New text on page 18

 ‘ … two reported a positive relationship between degree of latitude and melanoma mortality, suggesting that those living at more northerly latitudes, where there is less sunlight, experienced higher levels of melanoma mortality’

 Suggested revision

 ‘… two reported a positive relationship between degree of latitude and melanoma mortality, suggesting that those living at more northerly latitudes, where there is less UV in sunlight, experienced higher levels of melanoma mortality. Also ….?

Are the rationale for, and objectives of, the Systematic Review clearly stated?

Yes

Is the statistical analysis and its interpretation appropriate?

Yes

Are sufficient details of the methods and analysis provided to allow replication by others?

Yes

Are the conclusions drawn adequately supported by the results presented in the review?

Partly

Reviewer Expertise:

Richard McKenzie is an internationally recognised expert in the measurement, understanding, and implications of solar UV radiation. He wrote the initial review, and sought further insight from Ben Liley, a fellow atmospheric scientist with particular expertise in the analysis of UV dosimeter measurements of personal exposure. James Liley also previously contributed to the latter, but is now a medically-qualified biostatistician. He has contributed to the questions on statistics, and shared insight into the public health context of the study

We confirm that we have read this submission and believe that we have an appropriate level of expertise to confirm that it is of an acceptable scientific standard.

# Reviewer report: Reviewer response for version 1

**DOI:** 10.3310/nihropenres.15197.r36176

**Published:** 2025-09-09

**Authors:** Richard McKenzie, Ben Liley, James Liley

**Affiliations:** 1 National Institute of Water and Atmospheric Research Lauder Atmospheric Research Station (Ringgold ID: 563277), Lauder, Otago, New Zealand; 2 Mathematical Sciences, University of Durham, Durham, UK

This is a carefully conducted and well-written review that attempts to assess the mortality *risk* caused by overexposure to UV in sunlight, compared with the latter’s potential**** *benefit* in lowering mortality rates. The risk is widely understood to be from skin cancers, especially melanoma, whereas the benefit has been argued for other cancers and cardio-vascular disease (CVD).

 The inconclusive result is unsurprising, given the difficulty of measurement, the time lags involved, and confounding factors. The inhomogeneity of data sources between studies must also have been challenging.

 The authors conclude that no changes in public messaging are justified. Although that may be quite controversial, we find the data presented is a useful guide to current knowledge on the risks and benefits. It also provides a possible stepping stone for future work. We therefore recommend to satisfactorily addressing the concerns about the conclusions raised below.

 Major Points

 1. None of the studies cited were able to directly compare UV exposure with mortality rates. UV dosimeters to measure personal exposure have been available for a relatively short time, and it in any case remains impractical to measure total doses over long periods of time, let alone lifetimes. As a results, the studies all use proxies for UV exposure. The best of these use ambient UV (measured or calculated), while others use measures as simple as latitude of residence as a proxy. These last are particularly troublesome given modern holiday habits where much of the annual exposure may occur in places at much lower latitudes. Consequently, in many if not most cases it was considered that there was a very high or high risk of bias.

 2. The repeated statement that some of the papers are considered biased deserves more elaboration. Further info on how this bias is assessed would be useful for readers who aren’t familiar with the ‘ROBINS-E’ tool. In particular, we would find it helpful to have an impression of whether ROBINS-E primarily assesses authorial good practice, or fundamental bias; that is, if study 1 has a large unavoidable bias in which the authors did everything they could to mitigate it, and study 2 is probably minimally biased but has poor reporting and acknowledgement of potential confounders, which study would be flagged as high- or low- bias risk? This is particularly relevant in this context, since studies using (e.g.) latitude as a proxy for UV exposure will generally be more confounded/biased than (potential) studies using a direct measurement, even with authorial best practice.

 3. None of the “all-cause mortality’ or ‘CVD Mortality’ results had bias risks lower than ‘High’.  What is the difference between ‘High’ and ‘Very high’ risk? Of the three main categories, only the ‘All Cancer Mortality’ results had what look to be acceptably low bias risks. Our inclination would be to discount all studies for which the risk of bias is ‘High’ or ’Very high’. Perhaps the authors could attempt to address how that may change their conclusions? In particular (especially given point 2 above) are some types of studies in this area inevitably susceptible to bias?

 4. Nearly 40 percent of studies considered used proxies like latitude. These may be more valid for assessing non-skin-cancer risks, which may depend on lack of winter exposure because, whatever the behavioural advice, populations confined to mid and high latitudes in winter will never be able to access sufficient UV from sunlight to maintain optimal levels of vitamin D. However, the validity of latitude as a proxy is less obvious in the case of skin cancer. Our own research has shown that populations in both northern and southern New Zealand receive less than 5 percent of the available UV for most of the year (see here (1)). Given that small proportion, behavioural differences probably far outweigh effects of latitudinal gradients, especially in summer when the excess exposures probably occur. This should be mentioned.

 5. While the collective studies do not provide strong evidence for a non-zero effect of UV exposure on mortality, CVD mortality, etc., they do provide moderate evidence for the absence of a large effect. The authors may wish to mention this.

 6. Further to this, although the overall risks from sun exposure clearly outweigh the benefits in the case of melanoma, that does not appear to be true for other cancers or CVD.  The regression studies (e.g., Table 1) report a (perhaps troublingly) large number of studies (particularly by Grant -re vitamin D) showing benefits from sun exposure – even for melanoma in a few of the studies. For other cancers, most notably breast cancer, sunlight exposure may be beneficial. But for all cancers and for CVD, it is not easy to see whether the benefits outweigh the risks.

 Given the much lower overall mortality rate from melanoma compared with that from CVD and all other cancers, it is indeed worth considering whether health messaging should be changed. Our main concern is that, if current guidelines do not stratify advice by skin colour, we feel that, on the basis of these results, they should do so. Given the low incidence of melanoma among dark skinned people, the relatively low levels of UV in the UK, and the larger fraction of the population who now fit into that category, it’s not clear from the present analysis whether total mortality burden from excessive sunlight exceeds that from insufficient exposure to sunlight. We do recognize the difficulty of changing public health  messaging, and that a full analysis of information relevant to deciding policies is beyond scope, but the authors may wish to mention this.

 7. We also note that some countries already advise supplementation of vitamin D over winter months. By adopting such a strategy, there’s less need to consider changing public health messaging of “minimizing exposure to sunlight”. However, as the authors remind us, deficiency in vitamin D is not the only winter issue. Low UV may affect other pathways as well in ways that aren’t yet fully understood. The authors also remind us that there’s little solid evidence for an association between vitamin-D status and mortality from cancer and CVD.

 Given the health implications raised here and the relative importance of the various forms of cancer and CVD for different skin type, we feel that the statement that ‘ *no change in health messaging is justified’* may be premature. Perhaps it would be better to recommend a follow-up study using the same data and attempt to estimate whether the overall burden of death from cancer and CVD increases or decreases as a function of sun exposure, and to assess whether the conclusion for white skinned people is the same as that for those with pigmented skins. Such a follow-up study should probably ignore studies for which the risk bias is high or very high.

 8. The question motivating this study, “Should health advice on sun exposure be changed?”, indeed seems to have no clear answer from the effects on years of lost life. By the same token, the question might have been, “Do studies of YLL , at least from the dominant causes of heart failure and cancer, inform what advice should be given on sun exposure?” That question would also be answered in the negative, but with the opposite effect. Some discussion of this might be warranted.

 Another question that might be addressed is whether changed mortality is on its own a sufficient basis for the advice. Many immune disorders are affected, in incidence or progression, by sun exposure; type 1 diabetes, multiple sclerosis, rheumatoid arthritis, coeliac disease, Graves’ disease, psoriasis and psoriatic arthritis, IBD, etc. Even if they do not result in early death, any of these can have a profound impact on quality of life. Using a measure like disability-adjusted life-years or health-adjusted life-years would presumably entail as much work again, and presumably another paper, but the possibility of doing so might usefully be raised here.

 Minor Point:

 P9, near top. There may be confusion about units for radiation measurements in the cited papers. It’s reported that an increase of 100 kJ m ^-2^ of solar radiation is associated with a 1% increased risk. Is that over a lifetime, or a year, or a day? In any of those cases, a measurable effect seems unlikely because in a single day, the total dose from sunlight can be over 20,000 kJ m ^-2^, so the reported excess is less than 0.5% of peak values (or about 1% for peak winter values). Perhaps they meant UVB for which daily doses would be smaller by at least a factor of 500 (with peak noon values around 2 W m ^-2^), or erythemal UV which would be smaller by a factor of at least 3500 (with peak noon values around 0.3 W m ^-2^, UVI =12). Even for those quantities, a causative dose increase of 100 J m ^-2^ seems rather small, given that erythema doses of 70 SED (7000 J m ^-2^ can occur in a single day. The results of Chen later on the same page, which report increases in daily average UVB look overly sensitive given those noon values quoted above. It’s also puzzling to see that increased dose reported in average watts rather than joules. It would be more useful the percentage increase risks were provided as function of the *percentage* increase in UV exposure.

Are the rationale for, and objectives of, the Systematic Review clearly stated?

Yes

Is the statistical analysis and its interpretation appropriate?

Yes

Are sufficient details of the methods and analysis provided to allow replication by others?

Yes

Are the conclusions drawn adequately supported by the results presented in the review?

Partly

Reviewer Expertise:

Richard McKenzie is an internationally recognised expert in the measurement, understanding, and implications of solar UV radiation. He wrote the initial review, and sought further insight from Ben Liley, a fellow atmospheric scientist with particular expertise in the analysis of UV dosimeter measurements of personal exposure. James Liley also previously contributed to the latter, but is now a medically-qualified biostatistician. He has contributed to the questions on statistics, and shared insight into the public health context of the study

We confirm that we have read this submission and believe that we have an appropriate level of expertise to confirm that it is of an acceptable scientific standard, however we have significant reservations, as outlined above.

## References

[1] : Association of 25‐Hydroxyvitamin D3 Levels in Adult New Zealanders with Ethnicity, Skin Color and Self‐Reported Skin Sensitivity to Sun Exposure. *Photochemistry and Photobiology* .2011;87(5) : 10.1111/j.1751-1097.2011.00956.x 1173-1178 10.1111/j.1751-1097.2011.00956.x 21679191

# Response

**Published:** 2025-12-08

**Authors:** Tom Parkhouse

**Affiliations:** University of Bristol Medical School, Bristol, England, UK

Many thanks to the reviewers for these helpful and insightful suggestions. We have attempted to address each point below.

**1. None of the studies cited were able to directly compare UV exposure with mortality rates. UV dosimeters to measure personal exposure have been available for a relatively short time, and it in any case remains impractical to measure total doses over long periods of time, let alone lifetimes. As a results, the studies all use proxies for UV exposure. The best of these use ambient UV (measured or calculated), while others use measures as simple as latitude of residence as a proxy. These last are particularly troublesome given modern holiday habits where much of the annual exposure may occur in places at much lower latitudes. Consequently, in many if not most cases it was considered that there was a very high or high risk of bias.**

**Response: **We agree with the point raised here – no measures of exposure were direct and there were various issues with the proxy radiation measures. These issues are addressed in the discussion. However, our intention with this review was to be comprehensive and thorough in attempting to assess the effect of sunlight, therefore we felt it was beneficial to include these measures, though grouped separately in order to maintain that distinction.

**2. The repeated statement that some of the papers are considered biased deserves more elaboration. Further info on how this bias is assessed would be useful for readers who aren’t familiar with the ‘ROBINS-E’ tool. In particular, we would find it helpful to have an impression of whether ROBINS-E primarily assesses authorial good practice, or fundamental bias; that is, if study 1 has a large unavoidable bias in which the authors did everything they could to mitigate it, and study 2 is probably minimally biased but has poor reporting and acknowledgement of potential confounders, which study would be flagged as high- or low- bias risk? This is particularly relevant in this context, since studies using (e.g.) latitude as a proxy for UV exposure will generally be more confounded/biased than (potential) studies using a direct measurement, even with authorial best practice.**

**Response: **The ROBINS-E tool assesses both authorial good practice and fundamental bias. We have added a brief description of the tool in the methods, addressing the point raised here as well as listing the seven domains the tool covers:

 “ROBINS-E is a tool designed to evaluate the risk of bias in results of observational epidemiologic studies, covering aspects of design, conduct and reporting of the study. It assesses risk of bias across seven domains: confounding, measurement of the exposure, selection of participants, post-exposure interventions, missing data, measurement of the outcome and selection of the reported result.”

**3. None of the “all-cause mortality’ or ‘CVD Mortality’ results had bias risks lower than ‘High’.  What is the difference between ‘High’ and ‘Very high’ risk? Of the three main categories, only the ‘All Cancer Mortality’ results had what look to be acceptably low bias risks. Our inclination would be to discount all studies for which the risk of bias is ‘High’ or ’Very high’. Perhaps the authors could attempt to address how that may change their conclusions? In particular (especially given point 2 above) are some types of studies in this area inevitably susceptible to bias?**

**Response: **Looking at the primary outcomes – as the reviewers point out, no study has a RoB assessment lower than high for all-cause and all-CVD. So discounting all ‘high’ and ‘very high’ RoB results would leave no results for these two primary outcomes. For all-cancer, this would leave three studies in the forest plot and three in the table – the results of which are similar to those currently presented (i.e., most results in the direction of a beneficial effect). Two papers were assessed to be of very high RoB. For the Yang paper, this was due to it being a proxy measure of sunlight (frequency of annual sunburns) that was self-reported and had the potential for differential measurement error (e.g., those who never sunburnt less likely to mis-report frequency than those regularly sunburnt). For the Grant paper (ref 57), it was due to missing data being an issue for both the outcome and confounders (they excluded states with <40 deaths from lung cancer – a proxy for smoking).

**4. Nearly 40 percent of studies considered used proxies like latitude. These may be more valid for assessing non-skin-cancer risks, which may depend on lack of winter exposure because, whatever the behavioural advice, populations confined to mid and high latitudes in winter will never be able to access sufficient UV from sunlight to maintain optimal levels of vitamin D. However, the validity of latitude as a proxy is less obvious in the case of skin cancer. Our own research has shown that populations in both northern and southern New Zealand receive less than 5 percent of the available UV for most of the year (see here (1)). Given that small proportion, behavioural differences probably far outweigh effects of latitudinal gradients, especially in summer when the excess exposures probably occur. This should be mentioned.**

**Response: **We have increased the discussion around the applicability of the measures of exposure in the limitations section of the discussion. This includes mention of the issue you raise here regarding the lack of ambient UV received by individuals, as well as the validity of latitude when considering skin cancer. We also go on to discuss the difficulty of disentangling the effect of sunlight on the skin from the effect of associated confounders, given the lack of individual-level sunlight exposure measures:

 “This issue is further compounded for the proxy radiation measures, e.g., latitude. For example, a studies using personal dosimeters to investigate individual-level sun exposure in northern and southern latitudes in New Zealand showed that, regardless of latitude, people received less than 2% of ambient UVR exposure (Scragg et al, 2016 ). A finding which is echoed by a study in the UK showing that White people were exposed to ~2% ambient UVR, whilst those with darker skin types were exposed to even less, ~1% (Kift et al, 2013). As such, the validity of latitude as a proxy measure of radiation, especially when considering skin cancer, may be limited and results should be interpreted with caution.

 Furthermore, the fact that no study was conducted using a direct measurement of individual sunlight exposure makes it difficult to disentangle the specific effects of sunlight on the skin from various potential confounders. For example, it is feasible that people living in areas with higher levels of sunlight experience health benefits such as increased outdoor physical activity, better diet, and improved quality of life. Moreover, the effect of such confounders are likely to vary across region and/or country, further complicating the relationship.”

**5. While the collective studies do not provide strong evidence for a non-zero effect of UV exposure on mortality, CVD mortality, etc., they do provide moderate evidence for the absence of a large effect. The authors may wish to mention this.**

**Response: **We added the sentence “Overall, the current available evidence does not indicate a large effect of sunlight on mortality” to the conclusion.

**6. Further to this, although the overall risks from sun exposure clearly outweigh the benefits in the case of melanoma, that does not appear to be true for other cancers or CVD.  The regression studies (e.g., Table 1) report a (perhaps troublingly) large number of studies (particularly by Grant -re vitamin D) showing benefits from sun exposure – even for melanoma in a few of the studies. For other cancers, most notably breast cancer, sunlight exposure may be beneficial. But for all cancers and for CVD, it is not easy to see whether the benefits outweigh the risks.**

**Given the much lower overall mortality rate from melanoma compared with that from CVD and all other cancers, it is indeed worth considering whether health messaging should be changed. Our main concern is that, if current guidelines do not stratify advice by skin colour, we feel that, on the basis of these results, they should do so. Given the low incidence of melanoma among dark skinned people, the relatively low levels of UV in the UK, and the larger fraction of the population who now fit into that category, it’s not clear from the present analysis whether total mortality burden from excessive sunlight exceeds that from insufficient exposure to sunlight. We do recognize the difficulty of changing public health  messaging, and that a full analysis of information relevant to deciding policies is beyond scope, but the authors may wish to mention this.**

**Response: **We have increased discussion on the issues surrounding skin type/ethnicity and the lack of available data on this. We have added a new paragraph discussing the potential interaction between location and skin type (i.e., in low-UV countries, sunlight may benefit those with lighter skin, whilst in high-UV countries those with darker skin might benefit):

 “It is plausible that the relationship between geographical location and effect of sunlight exposure is moderated by skin type. Those with lighter skin may experience greater benefits of sunlight in higher latitude areas with typically low UV R. For example, in this review, the beneficial effect observed on all-cause and all-CVD mortality in northern Europe (Lindqvist et al, 2014; Stevenson et al, 2023). However, for those with darker skin, the benefits might be more pronounced in lower latitude, high UVR areas, as suggested by the beneficial effect observed for all-cancer mortality in Turkey (Altug & Kilçiksiz, 2020) and Hong Kong (Goggins et al, 2013). However, of these four studies, only Stevenson et al (2023) was specific in reporting the skin type/colour or ethnicity of their sample (restricting inclusion to only White participants). Therefore, such theories can only be made based on broad assertions about the skin type of large populations, and so ought to be made with caution.”

 Later, we have added a couple of sentences to highlight your point here that skin cancer was the only outcome with clear risks outweighing benefits, and that melanoma mortality is lower for those with darker skin:

 “Skin cancer mortality was the only outcome for which the evidence clearly suggested that the risks of sunlight exposure outweighed the benefits. Given that melanoma mortality is known to be lower in those with darker skin (Lopes, et al, 2021), this highlights the need for nuanced sun exposure guidance”

 Finally, we have added reference to the recent sun exposure guidance in Australia:

 “This, in turn, would help organizations responsible for sun safety messaging to provide more appropriate guidance for those with different skin types. For example, a recent summit of sun exposure experts in Australia promoted the use of sun safety advice that clearly distinguishes recommendations for people of different skin types (Neale et al, 2004).”

**7. We also note that some countries already advise supplementation of vitamin D over winter months. By adopting such a strategy, there’s less need to consider changing public health messaging of “minimizing exposure to sunlight”. However, as the authors remind us, deficiency in vitamin D is not the only winter issue. Low UV may affect other pathways as well in ways that aren’t yet fully understood. The authors also remind us that there’s little solid evidence for an association between vitamin-D status and mortality from cancer and CVD.**

**Given the health implications raised here and the relative importance of the various forms of cancer and CVD for different skin type, we feel that the statement that ‘no change in health messaging is justified’ may be premature. Perhaps it would be better to recommend a follow-up study using the same data and attempt to estimate whether the overall burden of death from cancer and CVD increases or decreases as a function of sun exposure, and to assess whether the conclusion for white skinned people is the same as that for those with pigmented skins. Such a follow-up study should probably ignore studies for which the risk bias is high or very high.**

**Response: **We agree, there is scope for various future research projects – both utilising the data we have extracted here, as well as novel primary research. We have now increased our discussion of suggestions for future work, before the concluding paragraph. One follow-up study we recommend is to develop a common unit of exposure measurement that the current radiation-based measures could be converted to – this would allow, at least, for statistical synthesis of the results, and would likely open the door to other useful projects.

**8. The question motivating this study, “Should health advice on sun exposure be changed?”, indeed seems to have no clear answer from the effects on years of lost life. By the same token, the question might have been, “Do studies of YLL , at least from the dominant causes of heart failure and cancer, inform what advice should be given on sun exposure?” That question would also be answered in the negative, but with the opposite effect. Some discussion of this might be warranted.**

**Another question that might be addressed is whether changed mortality is on its own a sufficient basis for the advice. Many immune disorders are affected, in incidence or progression, by sun exposure; type 1 diabetes, multiple sclerosis, rheumatoid arthritis, coeliac disease, Graves’ disease, psoriasis and psoriatic arthritis, IBD, etc. Even if they do not result in early death, any of these can have a profound impact on quality of life. Using a measure like disability-adjusted life-years or health-adjusted life-years would presumably entail as much work again, and presumably another paper, but the possibility of doing so might usefully be raised here.**

**Response: **Again, we agree that in principle the results presented in the review do not necessarily rule out the possibility that mortality is not a sufficient basis for sun safety advice. Although, the safety guidance is of course not only informed by mortality outcomes. There are many other outcomes that could, and should, be considered, such as incidence and quality of life. Unfortunately they were beyond the scope of the current review. However, as mentioned in reply to the point above, we have now increased discussion around future research ideas. As part of the suggested idea of converting results to a common unit of exposure, we mention that doing so could also prove useful for investigating other outcomes, such as incidence of disease.

**Minor point P9 - near top. There may be confusion about units for radiation measurements in the cited papers. It’s reported that an increase of 100 kJ m ^-2^ of solar radiation is associated with a 1% increased risk. Is that over a lifetime, or a year, or a day? In any of those cases, a measurable effect seems unlikely because in a single day, the total dose from sunlight can be over 20,000 kJ m ^-2^, so the reported excess is less than 0.5% of peak values (or about 1% for peak winter values). Perhaps they meant UVB for which daily doses would be smaller by at least a factor of 500 (with peak noon values around 2 W m ^-2^), or erythemal UV which would be smaller by a factor of at least 3500 (with peak noon values around 0.3 W m ^-2^, UVI =12). Even for those quantities, a causative dose increase of 100 J m ^-2^ seems rather small, given that erythema doses of 70 SED (7000 J m ^-2^ can occur in a single day. The results of Chen later on the same page, which report increases in daily average UVB look overly sensitive given those noon values quoted above. It’s also puzzling to see that increased dose reported in average watts rather than joules. It would be more useful the percentage increase risks were provided as function of the *percentage* increase in UV exposure.**

**Response: **We thank the reviewers for highlighting a typo here – that should have read 1,000 kJ/m ^2^. This has been corrected. In the case of all studies, we kept the units of measurement as reported in the paper.

# Reviewer report: Reviewer response for version 1

**DOI:** 10.3310/nihropenres.15197.r36115

**Published:** 2025-08-30

**Authors:** Carolyn Heckman

**Affiliations:** 1 Rutgers Cancer Institute, New Brunswick, USA

Important and timely topic and well-written paper.

 Title: Seeing the variety of types of exposures, I’m wondering whether “sunlight exposure” is the most appropriate term. You could also add something about the international sample.

 Abstract: Based on the Results, I suggest changing the final number of the articles in the Abstract and Figure 1 to 55.

 Lay Summary

1. Could be clarified. The first two sentences make it seem that burns rather than UV exposure cause all skin cancers.
2. Sunscreen is not mentioned.
3. I think something along the lines of “limits of available data” would be more accurate than “problems in how the studies were done.”
4. A reminder at the end about sun protection would seem appropriate.

Intro

1. Recommendations from the US National Cancer Institute might be more comparable to the other sources you note as opposed to the AAD.
2. I would include something about sunburns in childhood increasing risk for BCC too.

Methods

1. If no randomized trials were included, I would not mention that design. If they were, please report something about them, specifically.
2. Summarize the bias criteria.
3. Briefly describe the GRADE domains.
4. For studies that didn’t report race or ethnicity: Would it be appropriate to consider the race and ethnic composition of some of the study countries during the time(s) of data collection? E.g., studies conducted in China likely included mostly Asian participants. Or to consider findings for whites vs. non-whites, though admittedly less than ideal.
5. Would it be appropriate to weight the findings based on sample size, level of bias, null findings, and/or something else?

Results: p. 12, right paragraph – There seems to be an error in the reporting of the direction of the relationships.

 Discussion

1. There seem to be contradictory statements in the first paragraph.
2. The US population of Native Americans is quite small, and reporting for that group is notoriously poor. Those findings should be considered with caution.
3. Expand on the bias issues. What are the major confounders? One would be physical activity. Assuming that was considered in some studies, it might be worth including as an additional reporting variable.
4. The review separated studies by measure of UV, but as mentioned, risk is determined by available UV (e.g., latitude) and exposure to UV (e.g., behavior). Can these types of measures be combined somehow for studies in a similar/same place/time?
5. Wondering if it would be useful to include the tables/figures on specific cancers (other than skin) as supplements?
6. It would seem that the pancreatic cancer findings could be emphasized since they were mostly consistent.
7. The recent guidelines by Australians regarding tailored recommendations should be discussed in the Intro and/or Discussion and why those may be valid or not as compared to the current review.

Are the rationale for, and objectives of, the Systematic Review clearly stated?

Yes

Is the statistical analysis and its interpretation appropriate?

I cannot comment. A qualified statistician is required.

Are sufficient details of the methods and analysis provided to allow replication by others?

Yes

Are the conclusions drawn adequately supported by the results presented in the review?

Yes

Reviewer Expertise:

Behavioral skin cancer prevention.  My only statistical concern was how the male and female data were combined in some cases.

I confirm that I have read this submission and believe that I have an appropriate level of expertise to state that I do not consider it to be of an acceptable scientific standard, for reasons outlined above.

# Response

**Published:** 2025-12-08

**Authors:** Tom Parkhouse

**Affiliations:** University of Bristol Medical School, Bristol, England, UK

Many thanks for this thorough, helpful review of our paper. We have attempted to address your points and respond to each below.

**Title - Seeing the variety of types of exposures, I’m wondering whether “sunlight exposure” is the most appropriate term. You could also add something about the international sample.**

**Response: **We understand your point, though as sunlight exposure was the underlying effect we were interested in, and we have defined what this term encompasses, we feel that this is an appropriate title.

**Abst - Based on the Results, I suggest changing the final number of the articles in the Abstract and Figure 1 to 55.**

**Response: **Thank you. We have amended both the abstract and Fig 1 to make it clear that 55 articles were included in the narrative synthesis.

**Lay Summary**

**1. Could be clarified. The first two sentences make it seem that burns rather than UV exposure cause all skin cancers.**

**Response: **We have edited this sentence to make it more about UV exposure, whilst keeping it appropriate for a PLS:

 “Sunlight exposure of the skin can lead to the development of skin cancer”

**2. Sunscreen is not mentioned.**

**Response: **We have now included sunscreen:

 “Organizations providing advice on sun exposure say to wear sunscreen and covering clothing, and to stay in the shade between 11am and 3pm.”

**3. I think something along the lines of “limits of available data” would be more accurate than “problems in how the studies were done.”**

**Response: **We feel that limits of available data is a slightly different issue to the one we were referring to here (essentially RoB concerns). However, we have changed the wording to include the issue of data availability and to hopefully clarify the point:

 “However, there were issues with the amount of data available, as well as the quality of some of the data that was available…”

**4. A reminder at the end about sun protection would seem appropriate.**

**Response: **We have added to the final sentence to include this:

 “Currently, there is not strong enough evidence to alter sun exposure advice and so people should continue to follow the guidance.”

**Intro**

**1. Recommendations from the US National Cancer Institute might be more comparable to the other sources you note as opposed to the AAD.**

**Response: **Thank you. We have changed this reference from the AAD to the NCI.

**2. I would include something about sunburns in childhood increasing risk for BCC too.**

**Response: **We have added the statistic about childhood sunburns from the same review:

 “the risk of developing basal cell carcinoma (BCC), the most common form of non-melanoma skin cancer (NMSC), is 1.86 and 2.12 times greater with every five sunburns experienced as a child and as an adult, respectively”

**Methods**

**1. If no randomized trials were included, I would not mention that design. If they were, please report something about them, specifically.**

**Response: **No RCTs were included but we did search for them. We feel it is important to highlight that our search terms included RCTs, even if we failed to find any that met the inclusion criteria. Therefore, in the section of the results discussing the characteristics of the included studies, we have added the sentence “We did not identify any eligible randomized controlled trials.”

**2. Summarize the bias criteria.**

**Response: **We have provided some more information regarding the domains assessed with ROBINS-E:

 “ROBINS-E is a tool designed to evaluate the risk of bias in results of observational epidemiologic studies, covering aspects of design, conduct and reporting of the study. It assesses risk of bias across seven domains: confounding, measurement of the exposure, selection of participants, post-exposure interventions, missing data, measurement of the outcome and selection of the reported result.”

**3. Briefly describe the GRADE domains.**

**Response: **We have provided a brief description of the five GRADE domains:

 “The GRADE domains assess the risk of bias in the included studies (risk of bias); how consistent the results are within each outcome (inconsistency); how applicable the evidence is to the review question (indirectness); how precise the estimates are (imprecision); and the possibility of selective publication of results (publication bias).”

**4. For studies that didn’t report race or ethnicity: Would it be appropriate to consider the race and ethnic composition of some of the study countries during the time(s) of data collection? E.g., studies conducted in China likely included mostly Asian participants. Or to consider findings for whites vs. non-whites, though admittedly less than ideal.**

**Response: **Unfortunately, there was a lack of reporting of individual data regarding skin type/colour or ethnicity across the population level studies. Whilst we could use separate demographic information for countries/regions at the time of the study to infer race/ethnicity for some of the ecological studies, this is unfortunately beyond the scope of the current review and may not always be appropriate to perform as it would require broad assumptions. It also wouldn’t give us individual data, and therefore would not be appropriate to apply to case-control and cohort studies.

**5. Would it be appropriate to weight the findings based on sample size, level of bias, null findings, and/or something else?**

**Response: **As there is no statistical synthesis, any weighting would be challenging. However, we have added the number of participants included in the full cohort for all cohort studies with results for the primary outcomes in order to assist in interpretation. Where reported, we have also added the number of deaths among the cohort (relevant to each outcome). Sample sizes and considerations of power are less relevant to interpretation than are confidence intervals around estimated effect sizes, and we report these wherever they were available to us. We do not elaborate substantially on sample size differences, since in observational studies, larger is not necessarily better (as discussed, for example, by Egger et al (“Spurious precision? Meta-analysis of observational studies”. BMJ 1998; 316; https://doi.org/10.1136/bmj.316.7125.140).

**Results**

**p.12  - right paragraph – There seems to be an error in the reporting of the direction of the relationships.**

**Response: **We agree, this reporting was incorrect (and confusingly worded) – thank you for pointing this out. The text has now been corrected and hopefully the wording is clearer:

 “two found a positive relationship between degree of latitude and melanoma mortality, suggesting that those living at more northerly latitudes, where there is less sunlight, experienced higher levels of melanoma mortality.”

**Discussion**

**1. There seem to be contradictory statements in the first paragraph.**

**Response: **We have adapted the first sentence so that it now reads “a mixed message about the association between sunlight exposure and **all-cause** mortality risk.”

**2. The US population of Native Americans is quite small, and reporting for that group is notoriously poor. Those findings should be considered with caution.**

**Response: **We have added a note of caution to this result: “Although this result should be treated with caution as the Native American population is relatively small.”

**3. Expand on the bias issues. What are the major confounders? One would be physical activity. Assuming that was considered in some studies, it might be worth including as an additional reporting variable.**

**Response: **We have added a paragraph in the results regarding important confounders, with a brief overview of the most/least common factors controlled for:

 “The majority of results that were assessed for risk of bias were judged to be of some concern or high risk of bias. There were no results we judged to be low risk of bias across all domains, whilst some were assessed to be at very high risk of bias. The most common reasons for potential bias were uncontrolled confounding and concerns over the measurement of exposure. Concerns in the latter of these focused on the lack of direct measurement of personal sunlight  exposure, as is largely inevitable in large population-based studies, as well as the use of aggregated measures that may have misclassified individual exposure. In order to judge the risk of bias due to confounding, we prespecified a number of important confounding factors which should have ideally been controlled for, including age, sex/gender, ethnicity, smoking status, socioeconomic status, physical activity and altitude. No study included in the review controlled for all of these factors. The most commonly controlled for factors were age and sex/gender, followed by smoking. Looking at the primary outcomes, around half of the results controlled for race/ethnicity, though this was predominately achieved through restricting analysis to White participants. Physical activity was less well controlled for, whilst almost no studies controlled for altitude.”

 We have also added a paragraph in the discussion, addressing the difficulty in disentangling the effects of sunlight on the skin from confounders and lifestyle factors:

 “Furthermore, the fact that no study was conducted using a direct measurement of individual sunlight exposure makes it difficult to disentangle the specific effects of sunlight on the skin from various potential confounders. For example, it is feasible that people living in areas with higher levels of sunlight experience health benefits such as increased outdoor physical activity, better diet, and improved quality of life. Moreover, the effect of such confounders are likely to vary across region and/or country, further complicating the relationship.”

**4. The review separated studies by measure of UV, but as mentioned, risk is determined by available UV (e.g., latitude) and exposure to UV (e.g., behavior). Can these types of measures be combined somehow for studies in a similar/same place/time?**

**Response: **Although this would be an interesting extension to the findings, unfortunately there is insufficient individual data available to do this currently.

**5. Wondering if it would be useful to include the tables/figures on specific cancers (other than skin) as supplements?**

**Response: **NIHR Open requires all information that is necessary to follow the methods and results of the review to be included in the paper. Therefore, we are unable to send these to the supplement.

**6. It would seem that the pancreatic cancer findings could be emphasized since they were mostly consistent.**

**Response: **We have now added a sentence in the discussion to highlight the pancreatic cancer results:

 “In contrast, most articles examining the five cancers with the highest UK mortality rate (breast, prostate, lung, bowel and pancreatic cancer) found that higher levels of sunlight were associated with lower risks of mortality. In particular, for pancreatic cancer, where the available evidence was somewhat consistent in suggesting a beneficial effect of sunlight.”

**7. The recent guidelines by Australians regarding tailored recommendations should be discussed in the Intro and/or Discussion and why those may be valid or not as compared to the current review.**

**Response: **We have added reference to this guidance in the discussion, in relation to the paragraph addressing the lack of available evidence on skin type/race and the need for more tailored guidance:

 “Skin cancer mortality was the only outcome for which the evidence clearly suggested that the risks of sunlight exposure outweighed the benefits. Given that melanoma mortality is known to be lower in those with darker skin (Lopes, et al, 2021), this highlights the need for nuanced sun exposure guidance. However, approximately a quarter of the main articles included in this review were restricted to White populations. Around two thirds reported on the whole population, though given that a large number of these were conducted in Europe and North America, it is likely that the populations in those articles were predominantly White as well. In order to gain a more complete picture of the relationship between sunlight exposure and mortality, further studies investigating the impact of skin type/colour or ethnicity are warranted. This, in turn, would help organizations responsible for sun safety messaging to provide more appropriate guidance for those with different skin types. For example, a recent summit of sun exposure experts in Australia promoted the use of sun safety advice that clearly distinguishes recommendations for people of different skin types (Neale et al, 2004).”

# Reviewer report: Reviewer response for version 1

**DOI:** 10.3310/nihropenres.15197.r36173

**Published:** 2025-08-29

**Authors:** Douglas Brash

**Affiliations:** 1 Yale School of Medicine, New Haven, USA

Summary: The authors  used defined criteria to conduct an extensive search of the literature for epidemiological studies relating sunlight exposure to all-causes mortality and to more specific diesease endpoints. The results from these studies are then translated to a common effect metric and compared for each endpoint. The authors find that many studies indicate a beneficial effect of sunlight exposure for one endpoint or another, while other studies do not. The authors conclude that there is insufficient evidence to advise a change to current government and professional society advice on cautious sun-exposure.

 Critique: This prodigious effort addresses an important question raised by evidence that sunlight has various beneficial effects such as producing vitamin D and nitric oxide: How to balance these benefits against the fact that sunlight causes skin cancer, and balance against the pervasive advice to stay out of the sun and use sunscreen? An indicator of this need is that I and a colleague separately tried to get the American Academy of Dermatology to provide the references on which they based their sun protection recommendations, to no avail.

 The present report appears carefully done (I leave technical comments on the statistical methods and statistical-bias evaluations to other reviewers), and its summary of the literature is balanced rather than partisan. In using a Cochrane-like method, it progresses beyond earlier summaries such as Hoel et al, *Dermato-Endocrinology* 2016. I concur that the diversity in measurements used by the subject papers makes meta-analysis a fraught approach and I'm okay with the narrative approach plus Forest plots. The paper is therefore worthy of publication and *NIHR Open Research* is a suitable venue.

 I do have several comments and corrections, generally along the lines of the data not being as null as the authors state.

 Science comments:

 1. The graphical presentations in the Forest plots are misleading, as they underrepresent the beneficial effects. This is because the x-axis distance between 0.5 and 1.0 (reduction in harmful effect) is drawn as the same length as between 1.0 and 1.5 (harms), whereas 0.5 means a 2-fold reduction. The solution is to plot log2 of the effect size. Or at least plot 0.5 at the same distance from 1.0 as 2.0 is.

 2. The Lin[76] paper's measurement is correctly described in the text as a sunlight exposure measurement, but the Forest plot figures label it "biological damage". That is indeed reference 76's terminology, but it is inaccurate and, importantly, makes it harder to see the categorization of measurement metrics into Radiation exposure, Proxy for radiation exposure, and Behavioural. These entries should be relabeled "exposure" or something of the sort.

 3. For all-cause and all-CVD mortality (and less dramatically for others), the Lindqvist, Yang, and Goggins papers showed a beneficial effect sunlight, in striking contrast to other studies. The Discussion section notes that the first two were conducted in Sweden and so might reflect a sunlight benefit limited to low sun-exposure regions. It then worries that Goggins was conducted in Hong Kong, near the equator, so doesn't fit the concept. But if one factors in the darker skin in Hong Kong, a revised hypothesis would be that individuals with low sunlight penetration into skin might be the ones benefitting the most from sunlight. How to test that? Two approaches occur to me, not mutually exclusive.

 a) Make a 4-place table of # of studies finding a sunlight benefit vs harm X # conducted on populations with low vs ordinary sunlight presence in the skin. Then do a Fisher's exact test.

 b) It is of course conventional to plot effect sizes relative to the local control group, as done here. But that control group's absolute rate will vary between Sweden, UK, Hong Kong, and US, especially if there is indeed a beneficial or harmful effect of sunlight, and this change of the baseline might obscure the effect. How about plotting the absolute mortality rate in controls and +sun comparison groups (so two points per analysis instead of one)? In the case of a beneficial effect of sun, does sun exposure reduce the mortality rate in a low-sun region to that of a high-sun region? In which case one might not expect more sun in the high-sun region to have much beneficial effect.

 Another way to look at this is as a dose response that has an optimum (e.g. as vitamin-related effects often do). An increase in dose to the left of the minimal mortality peak (valley) will have one effect and the same amount of increase to the right of the valley will have the opposite effect.

 Item #3 is a suggestion, not a requirement for a revised ms.

 4. Abstract's Results summary. Using the word "inconclusive" will just tempt the reader to skip this paper and go on to the next one. The results are only inconclusive with regard to providing a single advisory on how much sunlight a person should seek. This situation is reasonable, as each biological effect of sunlight will have a different dose response (e.g. basal and squamous cell carcinomas have different UV dose responses), although it would have been nice if Nature had established a clear net benefit at some exposure.

 Unless the suggestions in #3 turn up something, I suggest saying that the literature's studies give mixed results that vary with the disease endpoint and with the geographical location of the population being studied, and it is therefore not possible to identify an ideal amount of sunlight exposure.

 That's different from inconclusive: I tell my students that an experiment that didn't support a beautiful hypothesis wasn't a failure, it was a success and the answer was "no".

 5. Some of the studies and meta-analyses cited in Hoel et al, *Dermato-Endocrinology* 2016 were not included here. Is it worth pointing out the basis for these omissions?

 Writing comments:

 p.5  "measured a single exposure", "measured two exposures"  What does this mean? Two actual exposures, eg two sunburns? Two types of exposure? Two measurement metrics for exposure?

 p.6 "Radiation" in the heading here and elsewhere. Sunlight is radiation, a thing. What is meant here is not the thing but amount of it to which the human is exposed, the "radiation exposure", "radiation dose", or more technically "radiation fluence". Maybe clearest to the average reader would be "Incident radiation" for these headings.

 p.9 All-cancer mortality overview. These results don't strike me as "the majority being in the direction of a beneficial association".

 p.12  "the results of six were in the direction of a beneficial effect of sunlight: two found that higher latitude (i.e. lower sunlight) was associated with lower melanoma" has to be read twice to find out it is not a contradiction. Perhaps re-word this.

Are the rationale for, and objectives of, the Systematic Review clearly stated?

Yes

Is the statistical analysis and its interpretation appropriate?

I cannot comment. A qualified statistician is required.

Are sufficient details of the methods and analysis provided to allow replication by others?

Yes

Are the conclusions drawn adequately supported by the results presented in the review?

Yes

Reviewer Expertise:

Biophysics, molecular genetics, and cell biology of sunlight-induced skin cancer; photobiology.

I confirm that I have read this submission and believe that I have an appropriate level of expertise to confirm that it is of an acceptable scientific standard, however I have significant reservations, as outlined above.

# Response

**Published:** 2025-12-08

**Authors:** Tom Parkhouse

**Affiliations:** University of Bristol Medical School, Bristol, England, UK

Many thanks for your review - your comments and suggestions were very helpful and have helped us to improve the paper. We have attempted to respond to each point below.

**1. The graphical presentations in the Forest plots are misleading, as they underrepresent the beneficial effects. This is because the x-axis distance between 0.5 and 1.0 (reduction in harmful effect) is drawn as the same length as between 1.0 and 1.5 (harms), whereas 0.5 means a 2-fold reduction. The solution is to plot log2 of the effect size. Or at least plot 0.5 at the same distance from 1.0 as 2.0 is.**

**Response: **Thank you for pointing out this oversight. We have now redrawn all forest plots using the exponential scale on the x-axis  in order to accurately represent the beneficial effects.

**2. The Lin[76] paper's measurement is correctly described in the text as a sunlight exposure measurement, but the Forest plot figures label it "biological damage". That is indeed reference 76's terminology, but it is inaccurate and, importantly, makes it harder to see the categorization of measurement metrics into Radiation exposure, Proxy for radiation exposure, and Behavioural. These entries should be relabeled "exposure" or something of the sort.**

**Response: **We agree with you that Lin et al inappropriately refer to “biological damage (BD/m2)”. The definition they provide is unclear and the use of the term is confusing; no biological damage has actually been measured. What it appears they have done is to obtain average July UVR data via TOMS in order to estimate individual levels of UV erythemal exposure, which in turn is divided into quartiles. As such, we believe it is correctly categorised as a ‘radiation’ measure. To avoid confusion, we have decided to remove mention of the units BD/m2 and instead labelled/referred to this exposure measure as quartiles of “Average July erythemal UVR.”

**3. For all-cause and all-CVD mortality (and less dramatically for others), the Lindqvist, Yang, and Goggins papers showed a beneficial effect sunlight, in striking contrast to other studies. The Discussion section notes that the first two were conducted in Sweden and so might reflect a sunlight benefit limited to low sun-exposure regions. It then worries that Goggins was conducted in Hong Kong, near the equator, so doesn't fit the concept. But if one factors in the darker skin in Hong Kong, a revised hypothesis would be that individuals with low sunlight penetration into skin might be the ones benefitting the most from sunlight. How to test that? Two approaches occur to me, not mutually exclusive.**

**a) Make a 4-place table of # of studies finding a sunlight benefit vs harm X # conducted on populations with low vs ordinary sunlight presence in the skin. Then do a Fisher's exact test.**

**b) It is of course conventional to plot effect sizes relative to the local control group, as done here. But that control group's absolute rate will vary between Sweden, UK, Hong Kong, and US, especially if there is indeed a beneficial or harmful effect of sunlight, and this change of the baseline might obscure the effect. How about plotting the absolute mortality rate in controls and +sun comparison groups (so two points per analysis instead of one)? In the case of a beneficial effect of sun, does sun exposure reduce the mortality rate in a low-sun region to that of a high-sun region? In which case one might not expect more sun in the high-sun region to have much beneficial effect.**

**Another way to look at this is as a dose response that has an optimum (e.g. as vitamin-related effects often do). An increase in dose to the left of the minimal mortality peak (valley) will have one effect and the same amount of increase to the right of the valley will have the opposite effect.**

**Item #3 is a suggestion, not a requirement for a revised ms.**

**Response: **Thank you for these carefully considered and insightful suggestions for further analyses. We have now added a paragraph in the discussion addressing the possibility that the relationship between location and effect of sunlight exposure is mediated by skin type. We highlight results in the review that support this theory:

 “It is plausible that the relationship between geographical location and effect of sunlight exposure is moderated by skin type. Those with lighter skin may experience greater benefits of sunlight in higher latitude areas with typically low UV R. For example, in this review, the beneficial effect observed on all-cause and all-CVD mortality in northern Europe (Lindqvist et al, 2014; Stevenson et al, 2023). However, for those with darker skin, the benefits might be more pronounced in lower latitude, high UVR areas, as suggested by the beneficial effect observed for all-cancer mortality in Turkey (Altug & Kilçiksiz, 2020) and Hong Kong (Goggins et al, 2013). However, of these four studies, only Stevenson et al (2023) was specific in reporting the skin type/colour or ethnicity of their sample (restricting inclusion to only White participants). Therefore, such theories can only be made based on broad assertions about the skin type of large populations, and so ought to be made with caution.”

 Whilst we agree that the suggestions you make for further analyses would make valuable contribution to the paper, unfortunately we don’t believe we have sufficient data to fully address suggestion a) at this stage. Given the lack of reporting on skin type/colour or ethnicity in the included studies, we don’t have the individual data required. As such, we would have to incorporate further population level data to map against each study – which is possible but comes with its own challenges and complications that are beyond the scope of this project currently. This is also the case for suggestion b), which may be more feasible to implement from the available data but would require a good deal of additional investigation. However, we believe these suggestions are absolutely worth exploring and as such have incorporated them into our suggestions for future research:

 “Firstly, there is a clear need for more research into the effects of sunlight on people with darker skin. The majority of studies were conducted in predominantly White populations, with many specifically limiting inclusion to White people only. Moreover, skin type/colour or ethnicity data ought to be more commonly reported in population level studies. This would allow for more insightful analyses of the available data. For example, allowing for further investigating into the hypothesis that those with darker skin benefit most from sunlight in high UVR areas.”

**4. Abstract's Results summary. Using the word "inconclusive" will just tempt the reader to skip this paper and go on to the next one. The results are only inconclusive with regard to providing a single advisory on how much sunlight a person should seek. This situation is reasonable, as each biological effect of sunlight will have a different dose response (e.g. basal and squamous cell carcinomas have different UV dose responses), although it would have been nice if Nature had established a clear net benefit at some exposure.**

**Unless the suggestions in #3 turn up something, I suggest saying that the literature's studies give mixed results that vary with the disease endpoint and with the geographical location of the population being studied, and it is therefore not possible to identify an ideal amount of sunlight exposure.**

**That's different from inconclusive: I tell my students that an experiment that didn't support a beautiful hypothesis wasn't a failure, it was a success and the answer was "no".**

**Response: **We have changed the term ‘inconclusive’ to ‘mixed’ which, as pointed out in the comment here, more accurately reflects the findings laid out in the following section of the abstract. We have also adapted the conclusion of the abstract to read “Findings from observational epidemiological studies of the association between sunlight exposure and mortality vary across different disease outcome and location being investigated. As such, the findings do not provide a strong rationale for changes to sun protection guidance.”

**5. Some of the studies and meta-analyses cited in Hoel et al, *Dermato-Endocrinology* 2016 were not included here. Is it worth pointing out the basis for these omissions?**

**Response: **We found this review in our initial scoping. Whilst it was a useful and relevant paper, we did not prioritise investigating their reference list as it was not a systematic review. However, we have since checked and do not believe we missed any studies that ought to have been included. Most were identified in our searches, some of which were included. Those that were excluded were most commonly either not measuring mortality; conducted on people with pre-existing disease; or were vitamin D studies. Of those that were not identified in our searches, having checked we do not believe any would meet the inclusion criteria.

**Writing comments**

**p.5 - "measured a single exposure", "measured two exposures"  What does this mean? Two actual exposures, eg two sunburns? Two types of exposure? Two measurement metrics for exposure?**

**Response: **We have amended this to ‘type(s) of exposure’

**p.6 - "Radiation" in the heading here and elsewhere. Sunlight is radiation, a thing. What is meant here is not the thing but amount of it to which the human is exposed, the "radiation exposure", "radiation dose", or more technically "radiation fluence". Maybe clearest to the average reader would be "Incident radiation" for these headings.**

**Response: **Where this category is first introduced, we have amended the text to “…categorised as measures of incident radiation (herein referred to as ‘radiation’).” This is hopefully more clear and accurate.

**p. 9 - All-cancer mortality overview. These results don't strike me as "the majority being in the direction of a beneficial association".**

**Response: **Whilst many of the results have wide confidence intervals (in the forest plot), or suggest very small associations (in the table), the overall direction of the results suggests a beneficial effect. Most of the studies report a point estimate, regression, or correlation result that favours sunlight – particularly when looking at the regression and correlation results reported in Table 1.

**p.12 - "the results of six were in the direction of a beneficial effect of sunlight: two found that higher latitude (i.e. lower sunlight) was associated with lower melanoma" has to be read twice to find out it is not a contradiction. Perhaps re-word this.**

**Response: **I think the wording was actually incorrect the first time – apologies and thank you for highlighting this. We have changed the wording to – “two found a positive relationship between degree of latitude and melanoma mortality, suggesting that those living at more northerly latitudes, where there is less sunlight, experienced higher levels of melanoma mortality.” Hopefully this is clearer.

# Reviewer report: Reviewer response for version 1

**DOI:** 10.3310/nihropenres.15197.r36172

**Published:** 2025-08-19

**Authors:** Leonardo Vinícius Monteiro de Assis

**Affiliations:** 1 University of Lübeck, Lübeck, Germany; 2 Chemistry and Molecular Biology, University of Gothenburg, Gothenburg, Västra Götaland County, Sweden

The manuscript from Parkhouse and colleagues is a systematic literature review that evaluated the effects of sunlight on human health. The authors used a solid literature review method, providing a clear methodological view on papers that were considered and discarded. The overall design of the study is well performed. Due to the mixed evidence in all parameters analyzed, the authors decided to write a narrative description of the data, which is pleasant and easy to follow. Overall, the authors’ conclusion is mixed in almost all parameters analyzed, except for the risk of skin cancer. On the other hand, the authors show evidence, albeit mixed, that sunlight has beneficial effects in many other parameters, including other forms of cancer. In the next lines, I have more specific suggestions to improve the manuscript:

1. Although the authors used the term sunlight, they rightly mentioned that other wavelengths are also included. However, the authors overfocused on UV, mostly UVB effects, but they did not mention the potential effects of visible light as a potential player in the negative and potentially beneficial effects of sunlight. In addition, the authors should discuss that, although UVB has been considered mostly a negative player, it only represents 2-5% of the solar spectrum. Conversely, visible light represents ca. 45% and infrared the remaining portion that reaches Earth. Therefore, I considered that this in itself is a biased reporting in the literature, where the effects of solar radiation have been attributed to UV (mostly UVB), leaving the impression that the other wavelengths (visible light and infrared) are innocuous. Several experimental studies show the potential negative effects of visible light on the skin, but when provided at a low level, visible light and its different wavelengths can have beneficial effects. The authors should address this issue, as it is critical to provide a well-balanced view on the sunlight effect.
2. Broadly defining the effect of sunlight on humans as either beneficial or negative is an oversimplification of the real situation. Sunlight effects are influenced by many factors, which, in essence, make the assessment of their effects a mighty task, which the authors elegantly tried to address. However, in my view, this could be better emphasized in the text, especially in the lay text. In addition, so many variables and covariates prevent the assessment of the sunlight effect per se. One suggestion to include is that sunlight has direct and indirect effects. Regarding the direct effects, for instance, one can argue about the consequences of UVB, UVA, and visible light on skin cells, contributing to skin cancer. However, the effects of sunlight on other parameters, such as CVD, breast, prostate cancer, etc, are likely due to indirect effects, such as increased physical activity/exercise, better life quality actions (e.g., diet) in this population. Therefore, while assessing the direct effects can be straightforward, evaluating the indirect effects of sunlight coexist with many variables that one cannot fully disentangle. Due to the complex nature of these effects, I suggest that the authors consider my suggestion and include a paragraph in the discussion.
3. Since the overall conclusions are already mixed, the authors should further emphasize that subgroup analysis for skin type is underpowered. I would further emphasize that such analyses and their conclusions are limited.
4. One should also consider that sunlight effects, either direct or indirect, can be influenced by the region/country, as the other variables, described in 2, are more diverse. This could be further discussed in the revised manuscript.
5. An interesting aspect of the study is that the authors classify the bias levels of the study. I find this very interesting. Considering that the evidence is mixed, could the authors, at least, suggest a few actions that could make future studies less biased and more standard? I understand that this is not the focus of this study, but it could serve as a building block for a future unifying methodological paper.

Are the rationale for, and objectives of, the Systematic Review clearly stated?

Yes

Is the statistical analysis and its interpretation appropriate?

Yes

Are sufficient details of the methods and analysis provided to allow replication by others?

Yes

Are the conclusions drawn adequately supported by the results presented in the review?

Yes

Reviewer Expertise:

I'm experienced in the fields of skin biology, circadian biology, photobiology, and metabolism.

I confirm that I have read this submission and believe that I have an appropriate level of expertise to confirm that it is of an acceptable scientific standard, however I have significant reservations, as outlined above.

# Response

**Published:** 2025-12-08

**Authors:** Tom Parkhouse

**Affiliations:** University of Bristol Medical School, Bristol, England, UK

Thank you for providing this thorough review. We have attempted to address your points and respond to each below:

**1. Although the authors used the term sunlight, they rightly mentioned that other wavelengths are also included. However, the authors overfocused on UV, mostly UVB effects, but they did not mention the potential effects of visible light as a potential player in the negative and potentially beneficial effects of sunlight. In addition, the authors should discuss that, although UVB has been considered mostly a negative player, it only represents 2-5% of the solar spectrum. Conversely, visible light represents ca. 45% and infrared the remaining portion that reaches Earth. Therefore, I considered that this in itself is a biased reporting in the literature, where the effects of solar radiation have been attributed to UV (mostly UVB), leaving the impression that the other wavelengths (visible light and infrared) are innocuous. Several experimental studies show the potential negative effects of visible light on the skin, but when provided at a low level, visible light and its different wavelengths can have beneficial effects. The authors should address this issue, as it is critical to provide a well-balanced view on the sunlight effect.**

**Response: **Thank you. We have highlighted your point regarding consideration to VL and IRR effects with respect to mortality, and have incorporated this within our paragraph that discusses the solar radiation measurements in the studies included in this review. We have also referenced the helpful paper you have cited. We have not specifically mentioned % of ambient radiation as effects are also dependent on factors including energy of the specific radiation (e.g. as would apply to a UVA vs UVB comparison), and this aspect would be rather technical to explain fully.  

 “Therefore, it is warranted to consider a broader range of solar radiation and its effects with respect to mortality, than UVB alone. Apart from UVR, this includes the VL and IRR which are also emitted by the sun, and reach and penetrate the skin where they may have biological effects. For example, experimental research has indicated that VL may contribute to skin cancer development as well as having potential beneficial effects (de Assis, et al 2021).”

https://doi.org/10.1016/j.jphotochemrev.2021.100403

**2. Broadly defining the effect of sunlight on humans as either beneficial or negative is an oversimplification of the real situation. Sunlight effects are influenced by many factors, which, in essence, make the assessment of their effects a mighty task, which the authors elegantly tried to address. However, in my view, this could be better emphasized in the text, especially in the lay text. In addition, so many variables and covariates prevent the assessment of the sunlight effect per se. One suggestion to include is that sunlight has direct and indirect effects. Regarding the direct effects, for instance, one can argue about the consequences of UVB, UVA, and visible light on skin cells, contributing to skin cancer. However, the effects of sunlight on other parameters, such as CVD, breast, prostate cancer, etc, are likely due to indirect effects, such as increased physical activity/exercise, better life quality actions (e.g., diet) in this population. Therefore, while assessing the direct effects can be straightforward, evaluating the indirect effects of sunlight coexist with many variables that one cannot fully disentangle. Due to the complex nature of these effects, I suggest that the authors consider my suggestion and include a paragraph in the discussion.**

**Response: **We have added a paragraph in the results regarding important confounders, with a brief overview of the most/least common factors controlled for:

 “The majority of results that were assessed for risk of bias were judged to be of some concern or high risk of bias. There were no results we judged to be low risk of bias across all domains, whilst some were assessed to be at very high risk of bias. The most common reasons for potential bias were uncontrolled confounding and concerns over the measurement of exposure. Concerns in the latter of these focused on the lack of direct measurement of personal sunlight  exposure, as is largely inevitable in large population-based studies, as well as the use of aggregated measures that may have misclassified individual exposure. In order to judge the risk of bias due to confounding, we prespecified a number of important confounding factors which should have ideally been controlled for, including age, sex/gender, ethnicity, smoking status, socioeconomic status, physical activity and altitude. No study included in the review controlled for all of these factors. The most commonly controlled for factors were age and sex/gender, followed by smoking. Looking at the primary outcomes, around half of the results controlled for race/ethnicity, though this was predominately achieved through restricting analysis to White participants. Physical activity was less well controlled for, whilst almost no studies controlled for altitude.”

 We have also added a paragraph in the discussion, elaborating on the point you make here to discuss the difficulty in disentangling the effects of sunlight on the skin from confounders and lifestyle factors:

 “Furthermore, the fact that no study was conducted using a direct measurement of individual sunlight exposure makes it difficult to disentangle the specific effects of sunlight on the skin from various potential confounders. For example, it is feasible that people living in areas with higher levels of sunlight experience health benefits such as increased outdoor physical activity, better diet, and improved quality of life. Moreover, the effect of such confounders are likely to vary across region and/or country, further complicating the relationship.”

**3. Since the overall conclusions are already mixed, the authors should further emphasize that subgroup analysis for skin type is underpowered. I would further emphasize that such analyses and their conclusions are limited.**

**Response: **We have added a sentence at the end of the paragraph on skin type subgrouping in the discussion to emphasise the limited conclusions due to lack of data:

 “Though, given the lack of available data on skin type/colour or ethnicity, any conclusions drawn from these studies are limited.”

**4. One should also consider that sunlight effects, either direct or indirect, can be influenced by the region/country, as the other variables, described in 2, are more diverse. This could be further discussed in the revised manuscript.**

**Response: **We hope the final sentence at the end of the new paragraph (quoted in point 2 above) adequately addresses the potential variations in confounders,  such as diet and quality of life, across regions.

**5. An interesting aspect of the study is that the authors classify the bias levels of the study. I find this very interesting. Considering that the evidence is mixed, could the authors, at least, suggest a few actions that could make future studies less biased and more standard? I understand that this is not the focus of this study, but it could serve as a building block for a future unifying methodological paper.**

**Response: **We have now included a section for future research suggestions. These cover the need for more accurate reporting of skin type/colour or ethnicity, as well as the broader need for more studies specifically looking at populations with darker skin types; the need for more studies utilising individual-level, personal sun exposure data; and the suggestion to focus on standardising the measurement of sunlight exposure, and the methods used to measure it.

# Reviewer report: Reviewer response for version 1

**DOI:** 10.3310/nihropenres.15197.r36178

**Published:** 2025-08-19

**Authors:** Rasha Shraim

**Affiliations:** 1 Trinity College Dublin, Dublin, Ireland

The authors of this study present an interesting and comprehensive systematic review on the effects of sun exposure on mortality. This is timely and useful research as this topic has been gaining interest among epidemiologists and other health researchers. The authors received input from a public advisory group and this is reflected positively in the clear and informative language of the summary. It is unfortunate that a meta-analysis could not be performed but the overall methods and approach are sound. My comments are below, mainly to improve the clarity of the reported results.

1. Most of the studies identified are reported as at ‘high’ or even ‘very high’ risk of bias. This merits the inclusion of some text in the results section describing the reasons why this is the case. The details are available in table S4 but considering that this is a concern for the majority of included studies, it’s worth including in the main text and, for example, mentioning the shared concern around confounding.
2. Related to the above, the authors do not report much on the covariates/other factors considered in these studies. It would be useful to know what these studies are generally adjusting for in their analyses or even *not* adjusting for. In some cases, the original studies stratified by sex or ethnicity so that is clear but in general what factors were considered? Again, some of this information is in table S4 but worth including in the main text, for example, the authors include an important note on smoking for one of the studies in all-cause mortality.
3. For cohort and case-control studies, some discussion on sample size is needed. The size range is wide (from the hundreds to the hundred thousands) and this should be considered in the interpretation of the results as it has important implications for statistical power.

Minor comments:

1. “…different skin types (three with Afro-Caribbean ancestry and one with very pale skin).” Please use a consistent descriptor, either ancestry or skin colour.
2. Data extraction methods indicate ‘funding source’ was collected but no further comments are available, were there any observations drawn from this data?
3. In the table footnotes, RoB is included as an abbreviation but not used in some tables and ‘b’ should be replaced with ‘β’ for consistency.
4. What was the specific continuous outcome measured for the linear regressions?
5. In the discussion, “…whilst there are long established risks associated with sunlight exposure in high UVR locations, the benefits of sunlight may possibly outweigh the harms in regions with a generally low UV index.” The possibility of interaction with population-level differences should also be mentioned (and this ties in with the next paragraph on differences between ethnicities).
6. In the discussion, “This, in turn, would allow organizations responsible for sun safety messaging to provide more nuanced guidance for those with different skin types.” A recent Australian study did this and would be an important reference to include here: [ Ref-1 : (Neale R, et al.,(2024) https://doi.org/10.1016/j.anzjph.2023.100117.]
7. Prior to their concluding remarks, it would be useful if the authors can include recommendations for future studies, especially considering the present high risk of bias and inconclusive results.

Are the rationale for, and objectives of, the Systematic Review clearly stated?

Yes

Is the statistical analysis and its interpretation appropriate?

Yes

Are sufficient details of the methods and analysis provided to allow replication by others?

Yes

Are the conclusions drawn adequately supported by the results presented in the review?

Partly

Reviewer Expertise:

Epidemiology, genetic epidemiology, sun exposure and vitamin D

I confirm that I have read this submission and believe that I have an appropriate level of expertise to confirm that it is of an acceptable scientific standard, however I have significant reservations, as outlined above.

## References

[1] : Balancing the risks and benefits of sun exposure: A revised position statement for Australian adults. *Australian and New Zealand Journal of Public Health* .2024;48(1) : 10.1016/j.anzjph.2023.100117 10.1016/j.anzjph.2023.100117 38350754

# Response

**Published:** 2025-12-08

**Authors:** Tom Parkhouse

**Affiliations:** University of Bristol Medical School, Bristol, England, UK

Many thanks for taking the time to provide this helpful and insightful review. We attempted to address all of your points and provide our responses below.

**1. Most of the studies identified are reported as at ‘high’ or even ‘very high’ risk of bias. This merits the inclusion of some text in the results section describing the reasons why this is the case. The details are available in table S4 but considering that this is a concern for the majority of included studies, it’s worth including in the main text and, for example, mentioning the shared concern around confounding.**

**Response: **We have added a paragraph to the results that discusses the overall risk of bias and some of the key reasons for the volume of high risk assessments (confounding and measurement of exposure):

 “The majority of results that were assessed for risk of bias were judged to be of some concern or high risk of bias. There were no results we judged to be low risk of bias across all domains, whilst some were assessed to be at very high risk of bias. The most common reasons for potential bias were uncontrolled confounding and concerns over the measurement of exposure. Concerns in the latter of these focused on the lack of direct measurement of personal sunlight  exposure, as is largely inevitable in large population-based studies, as well as the use of aggregated measures that may have misclassified individual exposure. In order to judge the risk of bias due to confounding, we prespecified a number of important confounding factors which should ideally have been controlled for, including age, sex/gender, ethnicity, smoking status, socioeconomic status, physical activity and altitude. No study included in the review controlled for all of these factors. The most commonly controlled for factors were age and sex/gender, followed by smoking. Looking at the primary outcomes, around half of the results controlled for race/ethnicity, though this was predominately achieved through restricting analysis to White participants. Physical activity was less well controlled for, whilst almost no studies controlled for altitude.”

**2. Related to the above, the authors do not report much on the covariates/other factors considered in these studies. It would be useful to know what these studies are generally adjusting for in their analyses or even *not* adjusting for. In some cases, the original studies stratified by sex or ethnicity so that is clear but in general what factors were considered? Again, some of this information is in table S4 but worth including in the main text, for example, the authors include an important note on smoking for one of the studies in all-cause mortality.**

**Response: **We have added a brief overview of the types of adjustment observed within the paragraph mentioned in the point above.

**3. For cohort and case-control studies, some discussion on sample size is needed. The size range is wide (from the hundreds to the hundred thousands) and this should be considered in the interpretation of the results as it has important implications for statistical power.**

**Response: **We have added the number of participants included in the full cohort for all cohort studies with results for the primary outcomes. Where reported, we have also added the number of deaths among the cohort (relevant to each outcome). Sample sizes and considerations of power are less relevant to interpretation than are confidence intervals around estimated effect sizes, and we report these wherever they were available to us. We do not elaborate substantially on sample size differences, since in observational studies, larger is not necessarily better (as discussed, for example, by Egger et al (“Spurious precision? Meta-analysis of observational studies”. BMJ 1998; 316; https://doi.org/10.1136/bmj.316.7125.140).

**Minor points**

**1. “…different skin types (three with Afro-Caribbean ancestry and one with very pale skin).” Please use a consistent descriptor, either ancestry or skin colour.**

**Response: **We have changed the wording to “three with dark skin and one with light skin”

**2. Data extraction methods indicate ‘funding source’ was collected but no further comments are available, were there any observations drawn from this data?**

**Response: **Funding was poorly reported in general. Where information was available we did not note anything that raised concerns. We have added a line in the discussion that points out the potential for conflict of interest in terms of publication bias:

 “Source of funding was poorly reported in general and we cannot exclude that conflict of interest was also associated with publication bias.”

**3. In the table footnotes, RoB is included as an abbreviation but not used in some tables and ‘b’ should be replaced with ‘β’ for consistency.**

**Response: **Thank you for pointing out this error. The final column in all tables is now abbreviated to RoB and b has been replaced by β in all table footnotes.

**4. What was the specific continuous outcome measured for the linear regressions?**

**Response: **The tables have been edited in order to add the specific outcome for all results, where possible, e.g., “all-cancer mortality rate per 100,000/year.”

**5. In the discussion, “…whilst there are long established risks associated with sunlight exposure in high UVR locations, the benefits of sunlight may possibly outweigh the harms in regions with a generally low UV index.” The possibility of interaction with population-level differences should also be mentioned (and this ties in with the next paragraph on differences between ethnicities).**

**Response: **We have added a paragraph in the discussion discussing the possibility that the relationship between location and effect of sunlight exposure is mediated by skin type. We highlight results in the review that support this theory:

 “It is plausible that the relationship between geographical location and effect of sunlight exposure is moderated by skin type. Those with lighter skin may experience greater benefits of sunlight in higher latitude areas with typically low UVR. For example, in this review, the beneficial effect observed on all-cause and all-CVD mortality in northern Europe (Lindqvist et al, 2014; Stevenson et al, 2023). However, for those with darker skin, the benefits might be more pronounced in lower latitude, high UVR areas, as suggested by the beneficial effect observed for all-cancer mortality in Turkey (Altug & Kilçiksiz, 2020) and Hong Kong (Goggins et al, 2013). However, of these four studies, only Stevenson et al (2023) was specific in reporting the skin type/colour or ethnicity of their sample (restricting inclusion to only White participants). Therefore, such theories can only be made based on broad assertions about the skin type of large populations, and so ought to be made with caution.”

**6. In the discussion, “This, in turn, would allow organizations responsible for sun safety messaging to provide more nuanced guidance for those with different skin types.” A recent Australian study did this and would be an important reference to include here: [ Ref-1 : (Neale R, et al.,(2024) https://doi.org/10.1016/j.anzjph.2023.100117.]**

**Response: **Thank you for highlighting this guidance. The paper has now been mentioned and referenced:

 “This, in turn, would help organizations responsible for sun safety messaging to provide more appropriate guidance for those with different skin types. For example, a recent summit of sun exposure experts in Australia promoted the use of sun safety advice that clearly distinguishes recommendations for people of different skin types (Neale et al, 2004).”

**7. Prior to their concluding remarks, it would be useful if the authors can include recommendations for future studies, especially considering the present high risk of bias and inconclusive results.**

**Response: **We have now included a section for future research suggestions. These cover the need for more accurate reporting of skin type/colour or ethnicity, as well as the broader need for more studies specifically looking at populations with darker skin types; the need for more studies utilising individual-level, personal sun exposure data; and the suggestion to focus on standardising the measurement of sunlight exposure, and the methods used to measure it.

# Reviewer report: Reviewer response for version 1

**DOI:** 10.3310/nihropenres.15197.r36177

**Published:** 2025-08-19

**Authors:** Richard Weller, Maria Teresa Brito, Andrew Stevenson, Jiayue Gu

**Affiliations:** 1 The University of Edinburgh College of Medicine and Veterinary Medicine, Edinburgh, Scotland, UK; 2 School of GeoSciences, University of Edinburgh, Edinburgh, UK

This is an important subject and we are delighted to see it reviewed and grateful to the authors for their work on this. Our research group is actively involved in research on the effects of UV/sunshine on systemic health and we do however have significant concerns over the data and wish to make some comments.

 1.    America and Europe are very different UV environments and cannot be combined. 

 We are concerned that not enough context has been given for where studies looking at the relationship between sunlight exposure were performed as this significantly affects response measures.  This factor alone accounts for most of the variability in the relationship between UV and major outcome measures.  For example, reduced all-cause and cardiovascular mortality with increased sunlight exposure are found by Lindqvist, Stevenson, Yang and Goggins.  Lindqvist, Stevenson and Yang studied relative sun exposure in white skinned populations living in northern Europe (Sweden and the UK 50 to 68° N).  Lin and He found increased all-cause and cardiovascular mortality associated with sun exposure, but these studies were of white skinned people living in the USA which is much closer to the equator (25 to 45° N) with significantly higher UV.  Goggins finds reduced all-cause and cardiovascular mortality with increased sunlight exposure in an ethnically Chinese population studied in Taipei and Hong Kong (22 and 25°N).

 Skin colour determines biological response to sunlight/UV and white European skin is an evolutionary adaptation to the low light environment in Europe with strong selective pressure being seen for white skinned gene variants (Wilde S, et al., (2014) [Ref: 1] **10.1073/pnas.1316513111**).  Similarly, strong functional constraints against MC1R variants coding for pale skin are seen in all African populations (Harding R, et al., (2000) [Ref:2] **https://doi.org/10.1086/302863**).  South of North Carolina, the United States are in African latitudes and thus UV environments, and any adverse effects of excess UV here bear little relevance to white skinned north European populations.  This fundamental biological and evolutionary understanding is not given enough weight in this review, where studies from all latitudes are analysed together rather than considering whether a population is matched to ambient sunlight exposure by skin type.  The relationship between cancer mortality and sunlight is similar to that of all-cause/CVS mortality.  The great majority of papers shown an inverse relationship between sunlight exposure and cancer mortality.  Again, Lin and He studying white skinned populations in the USA find increased cancer mortality.

 Studies of other populations, when adequately powered all find reduced mortality from cancers overall with increased UV exposure, despite increased skin cancer in white skinned subjects. These studies are all of populations living in a UV environment to which they are evolutionarily adapted.   Altug (Turkey), Chen (China), Fukuda (Japan), Goggins (Hong Kong) and Stevenson (White British) all find an inverse relationship between sunlight exposure and cancer mortality.  Lindqvist (Sweden) and Yang (Sweden) were underpowered.

 2.    Biological plausibility and dose response relationships.

 Not enough weight has been given to biological plausibility.  Lin’s paper looking at American populations identifies higher mortality with UV, yet mortality from Cancer, Respiratory disease, Stroke, Injury, and Diabetes all peaked in Quartile 3 and decreased in Quartile 4 (the highest exposure), which does not support a straightforward dose–response relationship between UV exposure and disease-specific mortality. Instead, it suggests a potential threshold or non-linear association, or possibly reflects methodological limitations, such as exposure misclassification, arbitrary cutoff selection, or residual confounding. The only neat dose-response relationship Lin showed is that UV decreased infectious disease. The most prevalent causes of cancer found by Lin were lung, liver and prostate cancer, even though such a link with UV has never been shown before (in fact, the opposite). Melanoma mortality was raised (as expected) but only in the highest quartile of UV exposure (equating to UV exposure in Algeria).  No mention is made of non-melanoma skin cancer mortality being raised with UV exposure, even though cutaneous SCC is undisputedly induced by UV exposure in a dose dependent fashion and can act as a positive control for UV exposure.

 By contrast, studies identifying all-cause mortality reductions with sunlight exposure in high latitude countries demonstrate a dose-response relationship (Lindqvist).  This is consistent with a sunlight-driven beneficial mechanism.

 3.    The authors include an old pre-print version of Stevenson et al. The analysis has been improved since publication and peer review and could represent among the higher quality studies in the review. For example:

 •    Domain 2 (was rated as high): risk of bias arising from measurement of the exposure. The exposure is now a direct measure of solar radiation rather than latitude (proxy).

 •    Domain 5 (was rated as high): risk of bias due to missing date. The new analysis was multiple imputation to impute missing values. Should be low now.

 •    Triangulation and use of negative control help with internal validity

 4.    We are concerned about the inclusion of studies that report only unadjusted correlations, as these are highly susceptible to bias. Including such studies alongside adjusted analyses may give the impression that they carry equal evidentiary weight. We suggest either excluding these studies based on a revised criterion (e.g., excluding studies that do not adjust for potential confounders) or clearly separating them into a distinct section or supplementary file.

 5.    Studies using different levels of evidence for sun exposure are treated equally.  For example, He’s paper relies on dermatologist recorded actinic skin damage, but this proxy exposure can be influenced by a myriad of other environmental factors, and as the authors admit was heavily confounded, being strongly correlated with lower education levels, deprivation, outdoor working and living in the American south. By contrast, Stevenson and Lindqvist record direct measures of sunlight exposure and these are then validated (Stevenson) against serum vitamin D levels to confirm their accuracy.  The authors should give more emphasis to the validation of sun exposure measures in the studies utilised, as this will influence what conclusions can be drawn from reliable data given the variability in measures within the field.

 6.    It would be helpful for the reader to better understand the evidence base if the review structured the evidence by study quality (perhaps by rating (lowest, middle, highest), or by ecological versus individual studies, if all studies in a section are rated the same quality). Also, by low and high sunlight environments – the authors could maybe colour code results according to low/high sun in graphs/tables. 

 Ecological studies, which analyse data aggregated at the group level (e.g. countries, regions), have different strengths and weaknesses compared to studies that examine individual-level data. Separating them allows for a more accurate assessment of the evidence and avoids the potential for ecological fallacy.

 The authors note results that are compatible with higher or lower risk (harmful or beneficial) when they are just not statistically significant. If an association is not significant – but near statistical significance – it makes sense to consider it in a harmful/beneficial direction. But when the point estimate is higher or lower than 1 and the CI is wide (high p-value), it should not be considered beneficial or harmful. In the Discussion section, the authors start by stating that half of the articles addressing their primary outcome reported a positive direction effect, and the other half a negative direction effect. However, they do not refer to the fact that several of them have confidence intervals that show the results to not be significant. Of those that do not span 1 in their confidence intervals, the majority of results reports benefits from sunlight exposure.

 Little emphasis was put on multivariate vs univariate analyses in the Results section. In an area as subject to confounding as this is, it would be helpful to refer to the number of factors that were controlled for in each paper. This will also influence the weight that can be attributed to studies’ results – a statistically significant result with inadequate adjustment for confounding is still not reliable.

 7.    It would be helpful to describe the relative contribution of mortality from CVD, cancer and specific cancers (maybe top 5 vs melanoma/NMSC) in the UK in the introduction since the paper is examining the benefits/harms of sun exposure; this provides context to the scope of the issue. 

 8.    The sun protection guidance focuses on skin cancer; however, in this review the evidence for sunlight exposure and skin cancer mortality is similar or lower to other cancers with a higher burden. This suggests that there is currently an overemphasis on skin cancer in the guidance and potential benefits of safe sun exposure should at least be acknowledged.

 Example, using the benefit/harm considered in text:

 -    Melanoma mortality risk: 34/46 74% of results harmful 

 -    Pancreatic: 9/11 = 81% beneficial 

 -    Bowel 22/31 = 71% beneficial 

 -    Lung cancer 7/12 = beneficial 58% 

 -    Breast 13/17 = beneficial 76% 

 -    All cancer 20/22 = beneficial 91% 

 9.    The authors rated most studies as high risk of bias. There should be a greater discussion about why the studies are high risk – e.g., what are the important confounders that are missing; direction of bias; consider reverse causation and misclassification. What are the authors recommendations for future cohort studies to reduce the risk of bias? This would help future researchers to design their studies to improve the evidence base.

 10.    While ROBINS-E was appropriately used to assess for risk of bias, would a tool like the Newcastle-Ottawa scale be useful to also assess the individual quality of the studies? This is especially relevant since certain articles were excluded from the ROBINS-E analysis due to the format of their findings; as such these would still undergo some form of quality assessment. This would help more substantial decisions to be made about what we can draw from the body of evidence.

 11.    The conclusion of this review is that ‘findings for overall mortality are too variable to provide a rationale for changes to sun protection guidance’.  As the authors feel that the evidence is in a state of equipoise, they advise no change to guidance; however, existing guidance is to actively limit sun exposure, and thus ‘no change’ in effect means continue avoiding sunlight. This would be appropriate if the evidence pointed firmly to overall harm induced by sunlight, but no such evidence is found. Our own interpretation of the data presented is that all studies from north Europe show benefits exceeding risks for sunlight exposure.  Only studies of white skinned populations in high UV environments show harm.  Of note, the Australian Skin and Skin Cancer Research Centre sun safety guidelines were revised in 2024 to actively acknowledge and consider sunlight’s health benefits and to tailor advise based on skin colour. As Australia is a markedly higher UV environment than the UK it is remarkable that they should take this approach, while we continue advising sunlight avoidance.

Are the rationale for, and objectives of, the Systematic Review clearly stated?

Yes

Is the statistical analysis and its interpretation appropriate?

Partly

Are sufficient details of the methods and analysis provided to allow replication by others?

Yes

Are the conclusions drawn adequately supported by the results presented in the review?

No

Reviewer Expertise:

Dermatology, photobiology, epidemiology, translational medicine

We confirm that we have read this submission and believe that we have an appropriate level of expertise to state that we do not consider it to be of an acceptable scientific standard, for reasons outlined above.

## References

[1] : Direct evidence for positive selection of skin, hair, and eye pigmentation in Europeans during the last 5,000 y. *Proceedings of the National Academy of Sciences* .2014;111(13) : 10.1073/pnas.1316513111 4832-4837 10.1073/pnas.1316513111 PMC397730224616518

[2] : Evidence for Variable Selective Pressures at MC1R. *The American Journal of Human Genetics* .2000;66(4) : 10.1086/302863 1351-1361 10.1086/302863 PMC128820010733465
